# Epiphytic common core bacteria in the microbiomes of co-located green (*Ulva*), brown (*Saccharina*) and red (*Grateloupia*, *Gelidium*) macroalgae

**DOI:** 10.1186/s40168-023-01559-1

**Published:** 2023-06-01

**Authors:** De-Chen Lu, Feng-Qing Wang, Rudolf I. Amann, Hanno Teeling, Zong-Jun Du

**Affiliations:** 1 Marine College, Shandong University, No. 180, Wenhua Xilu, Weihai, Shandong Province 264209 China; 2 Max Planck Institute for Marine Microbiology, Celsiusstraße 1, Bremen, 28359 Germany; 3 State Key Laboratory of Microbial Technology, Institute of Microbial Technology, Shandong University, Qingdao, 266237 China

**Keywords:** Algae, Bacteria, Biofilm, Biosynthetic gene cluster, *Gelidium*, *Grateloupia*, Macroalgae, Marine, Phycosphere, Microbiome, Polysaccharide utilization locus, *Saccharina*, *Ulva*

## Abstract

**Background:**

Macroalgal epiphytic microbial communities constitute a rich resource for novel enzymes and compounds, but studies so far largely focused on tag-based microbial diversity analyses or limited metagenome sequencing of single macroalgal species.

**Results:**

We sampled epiphytic bacteria from specimens of *Ulva* sp. (green algae), *Saccharina* sp. (brown algae), *Grateloupia* sp. and *Gelidium* sp. (both red algae) together with seawater and sediment controls from a coastal reef in Weihai, China, during all seasons. Using 16S rRNA amplicon sequencing, we identified 14 core genera (consistently present on all macroalgae), and 14 dominant genera (consistently present on three of the macroalgae). Core genera represented ~ 0.7% of all genera, yet accounted for on average 51.1% of the bacterial abundances. Plate cultivation from all samples yielded 5,527 strains (macroalgae: 4,426) representing 1,235 species (685 potentially novel). Sequencing of selected strains yielded 820 non-redundant draft genomes (506 potentially novel), and sequencing of 23 sampled metagenomes yielded 1,619 metagenome-assembled genomes (MAGs), representing further 1,183 non-redundant genomes. 230 isolates and 153 genomes were obtained from the 28 core/dominant genera. We analyzed the genomic potential of phycosphere bacteria to degrade algal polysaccharides and to produce bioactive secondary metabolites. We predicted 4,451 polysaccharide utilization loci (PULs) and 8,810 biosynthetic gene clusters (BGCs). These were particularly prevalent in core/dominant genera.

**Conclusions:**

Our metabolic annotations and analyses of MAGs and genomes provide new insights into novel species of phycosphere bacteria and their ecological niches for an improved understanding of the macroalgal phycosphere microbiome.

Video Abstract

**Supplementary Information:**

The online version contains supplementary material available at 10.1186/s40168-023-01559-1.

## Background

The term ‘macroalgae’ subsumes three major lineages: *Rhodophyta* (red algae), *Chlorophyta* (green algae) and *Phaeophyta* (brown algae) comprising approximately 12,000 species [1] that occur in coastal marine ecosystems worldwide. Macroalgae surfaces are colonized by bacteria and macroalgae-associated bacteria have co-evolved with macroalgae for roughly 1.6 billion years [2] with a complex and close relationship [3, 4]. The region of close algae-bacteria interactions is termed ‘phycosphere’ according to Bell and Mitchell (1972) [5]. The phycosphere microbiome is notably distinct from microbes of the surrounding seawater in terms of composition and functions [3, 4]. It supports the macroalgal host in essential functions, such as the morphological development [6] by the provision of growth factors [7], acclimation to environmental changes [8], release and settlement of algal spores [9], and the provision of vitamins and nutrients [7, 10]. Algal phycospheres also harbor potentially harmful bacteria, such as pathogens [11], or commensal bacteria that can degrade macroalgal tissues [12].

Macroalgae play an eminent role for maintaining high bioproductivity and biodiversity in coastal systems [13] and are thus of huge importance to various aspects of human life [14–16]. Compared to terrestrial plants, macroalgae have the benefits of higher growth rates, higher biomass yields, lower fiber, and higher polysaccharide contents [16]. Their combined biomass equals about 1,521 TgC yr^−1^ (range: 1,020-1,960 TgC yr^−1^) [17], and their ecological role thus parallels that of terrestrial plants. Macroalgae release 14 to 35% of their photoassimilated net primary production to the environment [18]. Some of this dissolved or aggregated particulate organic matter is rather recalcitrant and thus only slowly and partially degraded by marine bacteria. Such organic matter can sequester carbon for longer periods of time, as has been recently described for algal fucoidan [18]. However, most algal biomass is quickly remineralized by marine bacteria [19] and thereby routed back into the global carbon cycle.

Since macroalgae are usually sessile and predominantly inhabit coastal areas, they are subject to dynamic environmental changes, which in term affect their phycosphere community compositions [20]. Host morphology also plays a role, as has been shown with artificial algae of various shapes [3]. Such abiotic influences notwithstanding, phycosphere communities have shown to be also host-specific in various studies. For example, Lachnit et al. described both, seasonal variations and host specificities in the colonization patterns of three macroalgal species [21]. Different mechanisms have been proposed for host-specific colonization, such as a random occupation of phycosphere ecological niches by species with suitable adaptations, or the selection of functional genes on a community level [22, 23]. However, research is lacking for common core bacteria in different macroalgae in terms of taxonomy, representative genomes and ecophysiological functions.

Members of the following phyla dominate macroalgal phycospheres and are thus believed to be indispensable for proper phycosphere functioning: *Proteobacteria*, *Bacteroidota*, *Verrucomicrobiota*, *Planctomycetota*, *Firmicutes*, *Patescibacteria* and *Cyanobacteria* [3, 4, 10, 20–23]. Much less is known about these phycosphere bacteria than about those associated with terrestrial plants, particularly those of the rhizosphere. However, recent years have witnessed a growing interest in phycosphere bacteria of marine plants and algae that surpasses mere descriptions of microbial community composition, as is exemplified by recent studies of seaweed [24] and kelp microbiomes [10]. In particular the mechanisms that determine and maintain colonization patterns as well as the underlying genetic functions are of interest, not least because such functions bear the potential for useful industrial applications.

Two traits are prevalent among phycosphere bacteria, namely the potentials to degrade various algal polysaccharides and to produce a plethora of secondary metabolites. A substantial part of algal biomass consists of various diverse and complex polysaccharides. The primary polysaccharides in *Phaeophyta* are laminarins, fucoidans, cellulose and alginates [25], in *Chlorophyta* cellulose, xylans and ulvans [26, 27], and in *Rhodophyta* agars, carrageenans and galactans (including porphyran and furcellan) [15]. Many of these polysaccharides are anionic, sulfated and do not have equivalents in terrestrial plants [25]. In bacteria, the genes for the breakdown and take-up of polysaccharides are often co-located in dedicated polysaccharide utilization loci (PULs), in particular in the *Bacteroidota*. The capacity to degrade various land plant polysaccharides has been well studied in human gut *Bacteroidota* [26], and in some marine *Bacteroidota* targeting algal polysaccharides, e.g., alginate [28], laminarin [29, 30] and carrageenan [31]. However, a large-scale, systematic inventory of PULs of macroalgal phycosphere bacteria is as yet missing. Recent analyses have also shed light on the potential of marine bacteria to produce metabolites on a global scale, focusing either on planktonic bacteria [32] or marine biofilm-forming bacteria [33]. However, a comprehensive evaluation of the potential for secondary metabolite production of macroalgal phycosphere bacteria is lacking.

**Fig. 1:**
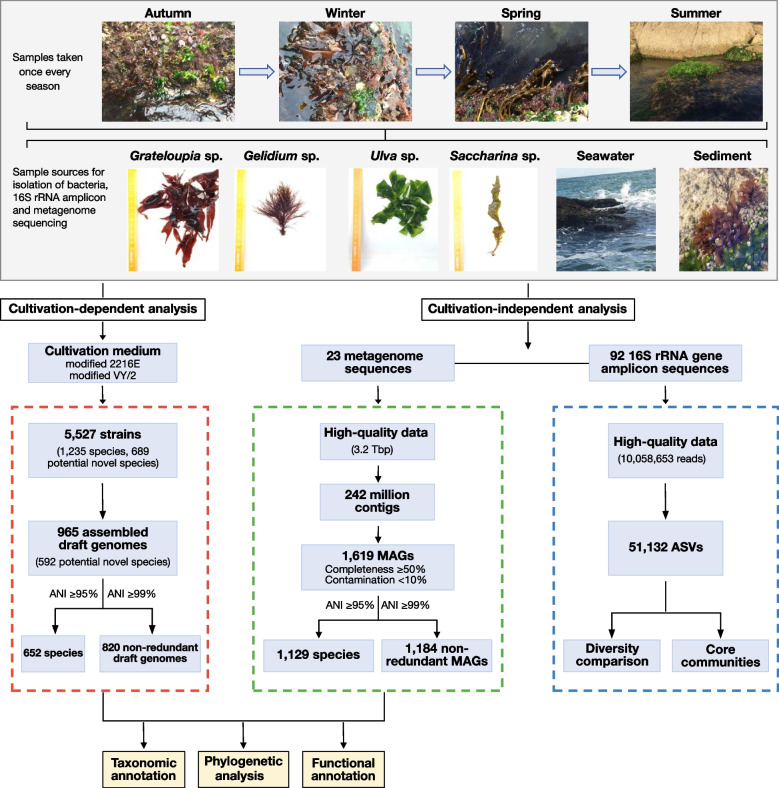
Study workflow. Samples were taken from a coastal reef in Weihai (China) once during each season. Four macroalgal species were sampled, plus sediment and seawater controls. Data analysis consisted of (i) the 16S rRNA gene tag pipeline (blue box), (ii) cultivation and draft genome sequencing of isolated strains (red box), and (iii) sequencing of community DNA with subsequent reconstruction of MAGs (green box)

In this study, we investigate phycosphere bacteria of four algal species: *Ulva* sp. (green algae), *Saccharina* sp. (brown algae), *Grateloupia* sp. and *Gelidium* sp. (both red algae). Samples were taken in spring, summer, winter and autumn together with seawater and sediment controls from a coastal reef at Weihai, China. We used a combination of 16S rRNA tag-based biodiversity analyses, extensive cultivation, as well as genome and deep metagenome sequencing in order to characterize and compare phycosphere communities, and in particular to identify common core genera (Fig. 1). We report a large number of cultured strains including novel core/dominant phycosphere strains, corresponding genomes, and insights into the potential of phycosphere bacteria to degrade algal polysaccharides and to synthesize bioactive secondary metabolites, some of which may control phycosphere community composition. The resulting comprehensive dataset of novel microbial species, their genomes and associated gene functions, represents a significant stepping stone towards a better understanding of the global ocean microbiome in general and macroalgal phycosphere bacteria in particular, and paves the way to functional studies on representative strains.

## Results

### All algae featured similar yet diverse phycosphere communities with notable seasonalities

Rarefaction curves of the 200 most abundant 16S rRNA ASVs (amplicon sequence variants) plateaued around 90% for most macroalgal and seawater samples. The top 20 ASVs alone accounted for close to 50% of the total abundance of the macroalgal samples, except for the *Saccharina* sp. brown algae summer samples and the two red algae species. The sediment samples were a different matter, as their rarefaction curves did not plateau, indicating higher overall diversities due to much higher numbers of rare taxa (Fig. S1b in Additional file 2).

In ASV α-diversity (richness) analyses, phycosphere samples exhibited similar overall diversities than seawater, but lower diversities than sediment samples, corroborating the rarefaction analyses (Fig. S1a in Additional file 2). Phycospheres were most diverse in summer except for *Gelidium* sp. (Fig. S1a in Additional file 2). Simpson’s diversity median values exceeded 0.8 for all habitats apart from *Saccharina* sp. in winter (0.5) due to high *Rubritalea* (*Verrucomicrobiota*) relative abundances (53.1% ± 30.7; see Discussion). Likewise, *Saccharina* sp. phycosphere communities had lower median Shannon diversity values (3.7 ± 1.8) than those from other macroalgae (4.3 ± 0.6) (Fig. S1a in Additional file 2).

**Fig. 2:**
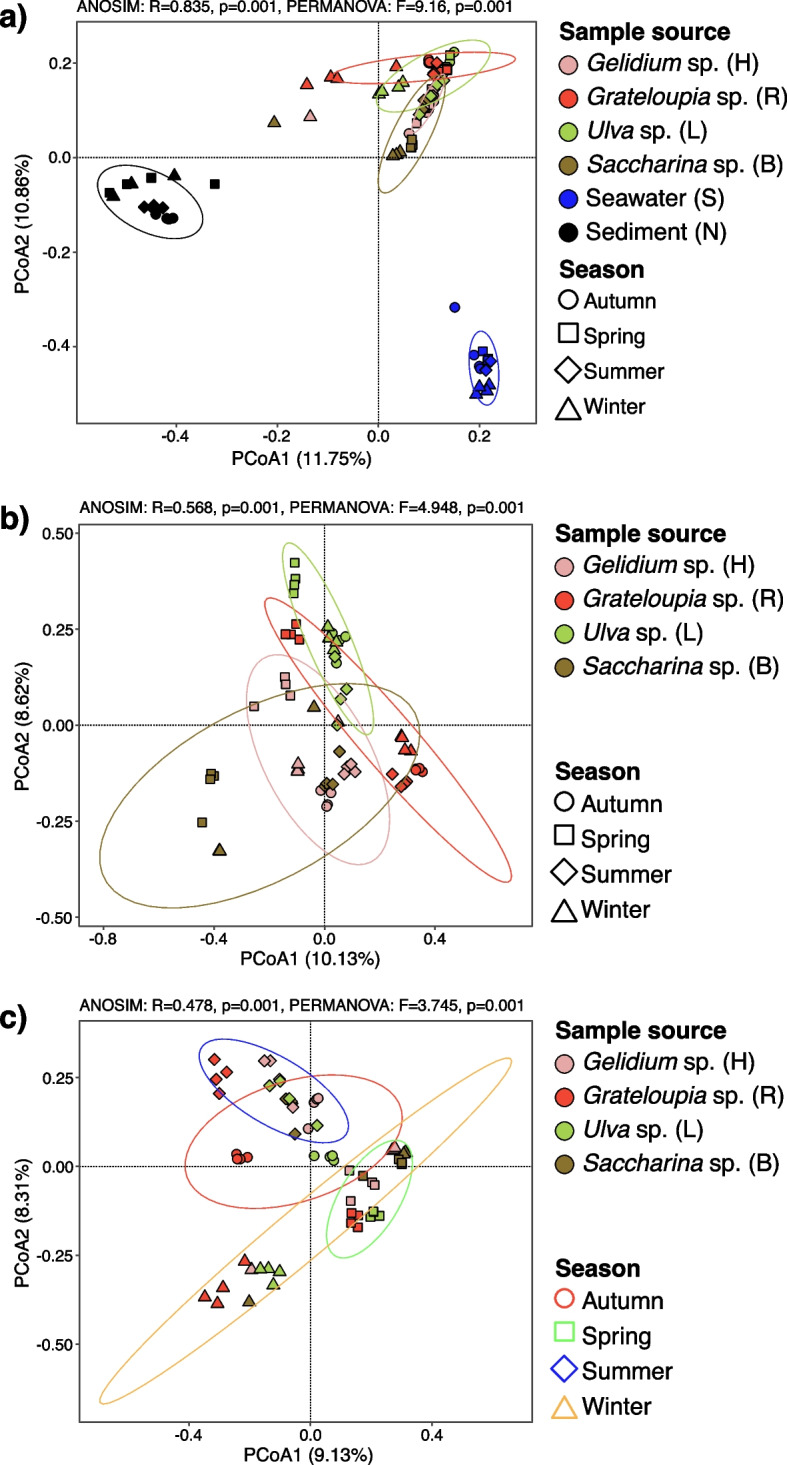
Principal coordinate analysis (PCoA) plots of Bray–Curtis similarities of samples and seasons calculated using unweighted UniFrac distances (each point corresponds to an individual sample). **a** macroalgal samples (*n* = 60), surrounding seawater (*n* = 15), and surrounding sediment (*n* = 17). **b** only macroalgal samples (*n* = 60). **c** only non-core macroalgal samples (*n* = 60). Details are provided in Additional file 3

Principal coordinate analysis (PCoA) of ASV β-diversity using the Bray–Curtis dissimilarity index revealed clustering by habitat (Fig. 2a), with phycosphere data clearly separated from sediment and seawater controls. Pairwise comparisons of only phycosphere samples, however, did not uncover significant differences, suggesting a considerable degree of shared taxa between the sampled macroalgal species (Fig. 2b). After removal of core taxa ASVs, i.e., of taxa occurring on all macroalgae (see Materials and methods), samples clustered more clearly according to season (Fig. 2c), indicating that non-core taxa contributed more to seasonal variation.

**Fig. 3:**
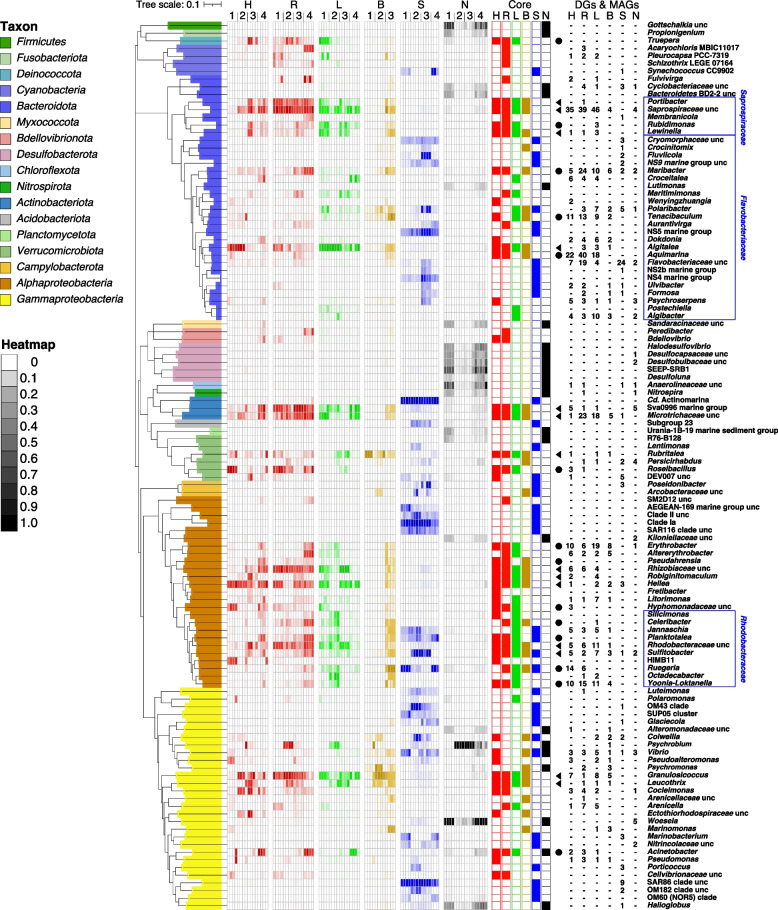
Phylogeny of 116 genera present in ≥ 85% of the samples of each habitat (four macroalgae plus sediment and seawater controls) with ≥ 1% relative abundance in at least one sample. Phylogenies were calculated using RAxML with 1,000 rapid bootstrap replicates based on similarities of full-length 16S rRNA gene sequences of the corresponding genera from SILVA NR Ref v138. Nomenclature: H = *Gelidium* sp., R = *Grateloupia* sp., L = *Ulva* sp., B = *Saccharina* sp., S = seawater, N = sediment, 1 = autumn, 2 = winter, 3 = spring, 4 = summer. Core phycosphere genera (present on all macroalgae) are highlighted by solid black triangles, and dominant phycosphere genera (present on three macroalgae) by solid black circles. Numbers in the six rightmost columns represent numbers of draft genomes (DGs) and MAGs obtained from all six habitats

The complete amplicon dataset comprised ASVs of 68 phyla, 56 of which were present on macroalgae (21,381 unique ASVs, Table S1 in Additional file 3). UniFrac UPGMA cluster analysis confirmed significant differences between the sediment, seawater and phycosphere habitats (Figs. 2, S3 in Additional file 2). The relative abundance of *Bacteroidota* in phycosphere samples was generally higher compared to seawater samples, which featured *Bacteroidota* abundances of up to 25.1% only in spring (Fig. S3 in Additional file 2). The sediment samples were even more distinct (Figs. 3, S3 in Additional file 2). Seasonal variations were obvious within all phycosphere communities (Figs. 3, S4 in Additional file 2). Samples from the same macroalgal species clustered for most seasons, particularly in the case of *Ulva* sp., *Grateloupia* sp. and *Gelidium* sp. in spring, suggesting particularly similar phycosphere communities (Figs. 2a, b, S3 in Additional file 2). Though differences among habitats became more apparent at the family and genus levels, there still was considerable consistency across macroalgal phycospheres (Figs. 3, S4 in Additional file 2).

### Phycospheres were dominated by few core phycosphere taxa

ASV analyses revealed that the majority of bacterial families in the phycospheres were represented by only one or two genera, while few, such as *Flavobacteriaceae* and *Rhodobacteraceae*, were more broadly represented (Figs. 3, S2 in Additional file 2, Table S1 in Additional file 3). This low overall evenness underscores that phycosphere communities were largely dominated by few abundant clades. Fourteen core genera from eight families (phyla *Proteobacteria*, *Bacteroidota*, *Verrucomicrobiota, Actinobacteriota*) were present on all macroalgae with ≥ 1% abundance in at least one of the samples (Fig. 3, Table 1, Table S1 in Additional file 3). *Sphingomonadaceae* and *Arenicellaceae* represented additional, diverse core families without any genus reaching ≥ 1% abundance in any sample (Fig. 3, Table S1 in Additional file 3). Core phycosphere genera comprised, on average, 1.4% of all phycosphere genera (*Gelidium* sp., 14/972, *Grateloupia* sp., 14/1,000, *Ulva* sp., 14/973 and *Saccharina* sp., 14/870), but accounted for on average 43.5% (*Gelidium* sp.), 53.9% (*Grateloupia* sp.), 58.3% (*Ulva* sp.) and 48.8% (*Saccharina* sp.) of all phycosphere bacteria (Table S1 in Additional file 3, Fig. S3, heatmap in Additional file 2). By comparison, the average relative abundances of these core phycosphere genera in seawater and sediment samples were only 5.7% and 1.5%, respectively (Table S1 in Additional file 3, Fig. S3, heatmap in Additional file 2). Fourteen additional genera were abundantly present in three of the four macroalgal species, hereinafter termed dominant genera (Fig. 3, Table 1). The relative abundances of all 28 prevalent genera varied in a similar fashion across seasons on all algae.

**Table 1:** List of the 14 core and 14 dominant phycosphere genera

**Taxa**	**Relative abundance (AVERAGE ± STDEV) (number of MAGs/draft genomes/cultured strains)**					
**Genus**	***Gelidium* sp. (H)**	***Grateloupia* sp. (R)**	***Ulva* sp. (L)**	***Saccharina* sp. (B)**	**Seawater (S)**	**Sediment (N)**
*Saprospiraceae* unc^a^	(3.96 ± 3.16) (35/-/-)	(10.15 ± 4.27) (39/-/-)	(4.28 ± 2.39) (47/-/-)	(1.79 ± 2.96) (4/-/-)	(0.08 ± 0.05) (-/-/-)	(0.2 ± 0.14) (4/-/-)
*Portibacter*^a^	(1 ± 0.96) (-/-/-)	(3.79 ± 1.59) (1/-/-)	(1.21 ± 1.13) (-/-/-)	(0.43 ± 0.72) (-/-/-)	(0.02 ± 0.03) (-/-/-)	(0.05 ± 0.04) (-/-/-)
*Lewinella*^a^	(0.96 ± 1.36) (1/-/-)	(0.65 ± 0.64) (1/-/-)	(2 ± 2.28) (3/-/-)	(0.88 ± 1.16) (-/-/-)	(0.01 ± 0.02) (-/-/-)	(0.03 ± 0.03) (-/-/-)
*Algitalea*^a^	(8.9 ± 11.7) (-/-/1)	(1.44 ± 1.17) (-/3/33)	(15.07 ± 13.44) (-/3/42)	(1.36 ± 0.62) (-/1/4)	(0.08 ± 0.06) (-/-/1)	(0.07 ± 0.07) (-/-/-)
*Microtrichaceae* unc^a^	(2.24 ± 1.95) (1/-/-)	(4.49 ± 2.57) (23/-/-)	(4.89 ± 6.18) (19/-/-)	(0.46 ± 0.58) (5/-/-)	(0.03 ± 0.04) (1/-/-)	(0.11 ± 0.1) (-/-/-)
Sva0996 marine group^a^	(1.97 ± 2.74) (-/-/-)	(9.23 ± 7.87) (5/-/-)	(4.89 ± 6.03) (1/-/-)	(0.64 ± 0.74) (1/-/-)	(0.27 ± 0.16) (5/-/-)	(0.28 ± 0.19) (-/-/-)
*Rubritalea*^a^	(2.06 ± 4.24) (1/-/-)	(0.62 ± 0.53) (-/-/-)	(1.02 ± 1.51) (1/-/-)	(24.37 ± 31.1) (1/-/-)	(0.13 ± 0.2) (-/-/-)	(0.04 ± 0.06) (-/-/-)
*Rhizobiaceae* unc^a^	(1.53 ± 1.64) (6/-/-)	(5.97 ± 3.34) (6/-/-)	(9.63 ± 14.93) (4/-/-)	(0.56 ± 0.83) (-/-/-)	(0.1 ± 0.11) (-/-/-)	(0.11 ± 0.13) (-/-/-)
*Robiginitomaculum*^a^	(1.22 ± 1.97) (2/-/1)	(0.56 ± 0.7) (-/-/-)	(0.32 ± 0.59) (4/-/-)	(0.38 ± 0.49) (-/-/-)	(0.01 ± 0.02) (-/-/-)	(0.01 ± 0.01) (-/-/-)
*Hellea*^a^	(7.45 ± 5.81) (-/1/2)	(3.08 ± 2.19) (-/-/-)	(4.69 ± 3.82) (2/-/-)	(0.49 ± 0.65) (2/-/-)	(0.11 ± 0.1) (3/-/-)	(0.11 ± 0.13) (-/-/-)
*Rhodobacteraceae* unc^a^	(2.32 ± 3.34) (9/-/-)	(3.99 ± 1.68) (8/-/-)	(3.45 ± 3.23) (13/-/-)	(0.49 ± 0.55) (1/-/-)	(0.19 ± 0.11) (7/-/-)	(0.1 ± 0.06) (3/1/-)
*Sulfitobacter*^a^	(0.68 ± 0.7) (2/3/26)	(0.97 ± 0.7) (1/1/18)	(0.64 ± 0.53) (-/7/39)	(1.04 ± 0.97) (-/3/38)	(4.52 ± 5.11) (1/-/2)	(0.07 ± 0.07) (-/2/17)
*Granulosicoccus*^a^	(7.55 ± 12.59) (5/2/5)	(7.84 ± 7) (1/-/1)	(4.32 ± 3.13) (8/-/-)	(11.65 ± 13.11) (4/1/4)	(0.13 ± 0.08) (-/-/1)	(0.22 ± 0.15) (-/-/-)
*Leucothrix*^a^	(1.69 ± 2.39) (-/-/-)	(1.13 ± 1.33) (1/-/-)	(2.81 ± 4.56) (1/-/-)	(3.1 ± 3.31) (1/-/2)	(0.04 ± 0.05) (-/-/-)	(0.05 ± 0.08) (-/-/-)
*Truepera*^b^	(2.12 ± 3.68) (-/-/-)	(1.74 ± 1.9) (-/-/-)	(2.91 ± 5.18) (-/-/-)	(0.15 ± 0.18) (-/-/-)	(0.01 ± 0.01) (-/-/-)	(0.18 ± 0.12) (-/-/-)
*Rubidimonas*^b^	(0.69 ± 0.82) (-/-/-)	(0.71 ± 0.75) (-/-/-)	(2.08 ± 1.98) (3/-/-)	(0.28 ± 0.36) (-/-/-)	(0.01 ± 0.01) (-/-/-)	(0.01 ± 0.02) (-/-/-)
*Maribacter*^b^	(0.83 ± 0.65) (4/1/21)	(1.41 ± 1.32) (6/18/81)	(0.31 ± 0.17) (2/8/27)	(1.13 ± 1.48) (1/5/31)	(0.02 ± 0.02) (-/2/2)	(0.12 ± 0.08) (-/2/10)
*Tenacibaculum*^b^	(0.75 ± 1.76) (-/11/27)	(0.09 ± 0.08) (1/12/46)	(0.31 ± 0.35) (2/7/56)	(3.37 ± 3.01) (1/1/6)	(0.06 ± 0.08) (-/-/1)	(0.03 ± 0.05) (-/-/14)
*Aquimarina*^b^	(0.29 ± 0.52) (5/17/87)	(0.49 ± 0.7) (3/37/236)	(0.05 ± 0.08) (8/10/38)	(0.37 ± 0.44) (-/-/14)	(0.01 ± 0.01) (-/-/1)	(0.03 ± 0.04) (-/-/1)
*Roseibacillus*^b^	(6.08 ± 10.53) (3/-/-)	(4.57 ± 4.83) (1/-/-)	(0.35 ± 0.79) (-/-/-)	(0.05 ± 0.06) (-/-/-)	(0.03 ± 0.02) (-/-/-)	(0.07 ± 0.04) (-/-/-)
*Erythrobacter*^b^	(0.41 ± 0.73) (-/10/60)	(0.47 ± 0.62) (-/6/40)	(0.53 ± 0.73) (5/14/53)	(0.36 ± 0.56) (1/7/22)	(0 ± 0.01) (-/-/1)	(0.03 ± 0.03) (-/1/15)
*Pseudahrensia*^b^	(0.66 ± 0.82) (-/-/1)	(0.51 ± 0.3) (-/-/-)	(0.27 ± 0.23) (-/-/-)	(0.63 ± 0.86) (-/-/-)	(0.02 ± 0.02) (-/-/1)	(0.09 ± 0.05) (-/-/-)
*Hyphomonadaceae* unc^b^	(2.41 ± 3.1) (3/-/-)	(0.65 ± 0.47) (-/-/-)	(0.55 ± 0.99) (-/-/-)	(0.25 ± 0.37) (-/-/-)	(0.03 ± 0.04) (-/-/-)	(0.01 ± 0.01) (-/-/-)
*Celeribacter*^b^	(0.23 ± 0.38) (-/-/-)	(1.43 ± 1) (-/-/-)	(1.21 ± 1.71) (1/-/-)	(0.98 ± 1.13) (-/-/-)	(0.03 ± 0.04) (-/-/7)	(0.02 ± 0.03) (-/-/1)
*Planktotalea*^b^	(0.61 ± 1.02) (-/-/1)	(1.41 ± 1.22) (-/-/-)	(1.28 ± 1.47) (-/-/-)	(0.72 ± 0.87) (-/-/-)	(1.79 ± 1.83) (-/-/1)	(0.05 ± 0.08) (-/-/1)
*Yoonia-Loktanella*^b^	(0.17 ± 0.3) (-/10/28)	(0.43 ± 0.39) (2/13/62)	(0.81 ± 1.16) (1/10/69)	(1.01 ± 2.01) (1/3/24)	(0.03 ± 0.03) (-/-/7)	(0 ± 0.02) (-/-/7)
*Ruegeria*^b^	(0.17 ± 0.25) (-/14/63)	(0.35 ± 0.33) (-/6/56)	(1.04 ± 0.91) (-/-/11)	(3.57 ± 4.87) (-/-/12)	(4.15 ± 4.23) (-/-/1)	(0.18 ± 0.13) (-/-/40)
*Acinetobacter*^b^	(3.62 ± 6.39) (1/1/1)	(1.78 ± 3.52) (1/2/4)	(5.06 ± 15.95) (1/-/3)	(0.03 ± 0.03) (-/-/2)	(0.12 ± 0.09) (-/-/-)	(0.41 ± 0.31) (-/-/3)

^a^Genus represents a core phycosphere genus

^b^Genus represents a dominant phycosphere genus

### Strains of 230 species from 16 abundant core and dominant phycosphere genera

Cultivation yielded in total 5,527 strains (macroalgae: 4,426). Clustering of their 16S rRNA gene sequences revealed that they represent 1,235 species (98.7% identity criterion) from 444 genera (94.5% identity criterion), including 968 species from macroalgae (Table S2 in Additional file 3). Almost two-thirds of the species were only isolated once (42.1%) or twice (19.3%). According to 16S rRNA amplicon analysis, about half of the macroalgal strains (2,492) exhibited ≥ 2% abundance in at least one macroalgal sample (Fig. S5 in Additional file 2, Table S2 in Additional file 3). As in 16S rRNA gene amplicon analysis, taxonomy patters of the isolated strains were more similar among macroalgal samples than between these and the sediment and seawater samples (Fig. S6 in Additional file 2).

We compared the 16S rRNA sequences of all strains with the 16S rRNA gene amplicon data representing 51,132 bacterial ASV nodes (Table S1 in Additional file 3). At a ≥ 98.7% identity criterion, 851 of the strains matched 787 ASVs (Table S2 in Additional file 3), with 618 strains matching a single ASVs, and 233 with one-to-many assignments to 169 additional ASVs. At a 97% identity criterion, a mean cultivability of 18.1% was obtained for macroalgal phycosphere species vs. 6.3% and 1.5% for seawater and sediments, respectively. Consequently, CFU numbers obtained from macroalgal samples (5.6 to 5.8 × 10^5^ CFU g^−1^ on average) were two to three orders higher than those from seawater and sediment samples, respectively (Fig. S7 in Additional file 2).

**Fig. 4:**
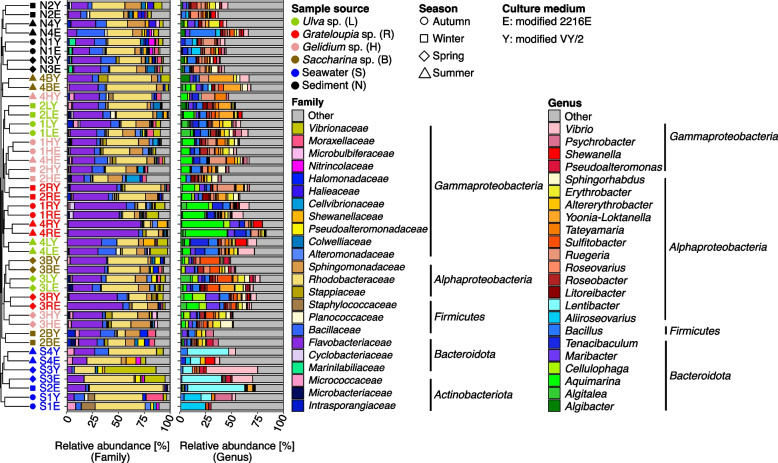
Cultivable phycosphere bacteria depending on macroalgal host, season and culture medium. Samples were grouped by weighted UniFrac distances using Ward linkage (dendrogram). Mean community compositions of the top 20 taxa are shown for family and genus levels

The strains included 735 novel species (577 from macroalgae). Proportions were highest among *Bacteroidota* (62.6%), *Proteobacteria* (53.6%), *Actinobacteriota* (16.1%), *Firmicutes* (7.8%), *Campylobacterota* (100%) and *Verrucomicrobiota* (100%) (Table S2 in Additional file 3). Without consideration of 29 strains with incomplete taxonomies, in total 230 species (1,556 strains) were representatives of 6/14 core and 10/14 dominant phycosphere genera (*Algitalea*, *Granulosicoccus, Hellea, Sulfitobacter*, *Leucothrix*, *Robiginitomaculum,* and *Maribacter*, *Tenacibaculum*, *Aquimarina*, *Erythrobacter*, *Planktotalea*, *Yoonia*-*Loktanella*, *Ruegeria*, *Acinetobacter*, *Pseudahrensia*, *Celeribacter*) (Fig. 4). In particular, the strains of *Granulosicoccus* (11), *Hellea* (2), *Leucothrix* (2) and *Robiginitomaculum* (1) are noteworthy, since members of these highly abundant phycosphere genera remain difficult to cultivate [6, 10, 12].

**Fig. 5:**
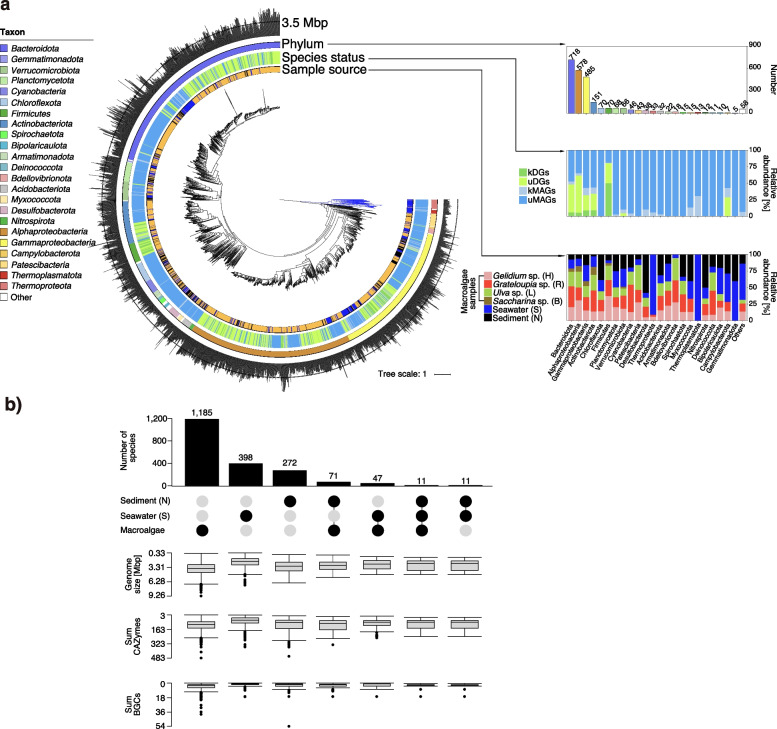
Metagenome-assembled genomes (MAGs) and draft genomes (DGs). **a** Phylogenomic tree of all 2,584 bacterial MAGs and DGs based on protein sequences of 43 universal single-copy genes with circles representing (inside to outside): (i) sample source and origin of the MAGs and DGs (relative proportions), (ii) known and unknown MAGs and DGs within the most abundant bacteria taxa with ≥ 5 genomes [state: unknown MAGs (uMAGs), known MAGs (kMAGs), unknown draft genomes (uDGs), and known draft genomes (kDGs)], (iii) GTDB phylum classification and absolute (redundant) numbers of MAGs and DGs obtained for each phylum, (iv) genome size (the tree was constructed using anvi’o v6.2 and visualized in iTOL v6.5.6). Total number of genomes from each sample: *Gelidium*: 539; *Grateloupia*: 609; *Saccharina*: 151; *Ulva*: 502; seawater: 469; sediment: 314. **b** Number of species-level MAGs and DGs that were either unique to or shared by sampled habitats. Vertical bars represent numbers of species shared between the study sets indicated by black dots in the lower panel

### Large numbers of draft genomes and MAGs from phycosphere bacteria, including novel species

Based on 16S rRNA sequence similarity, we selected 965 (macroalgae: 864) strains for draft sequencing, including 550 redundant novel species and 42 redundant novel genera (Tables S2, S3 in Additional file 3). Comparisons to 14,131 available published reference genomes [34] revealed that the obtained draft genomes corresponded to 652 species (95% ANI, 65% alignment) represented by 820 non-redundant DGs (99% ANI), including genomes of 399 (macroalgae: 342) novel species, as well as genomes of 246 (macroalgae: 221) species complementing validly described species not yet represented by genomes. From all metagenomes we obtained 1,619 (macroalgae: 936) MAGs with ≥ 50% completeness and < 10% contamination estimates. These corresponded to 1,129 species (95% ANI) represented by 1,184 non-redundant MAGs (99% ANI) (Fig. 1).

In total 961 DGs and 545 MAGs had > 90% completeness and < 5% contamination estimates, but did not fulfill MIMAG ‘high-quality’ criteria [35] due to 482 lacking complete rRNA gene operons. However, they did adhere to the ‘nearly complete’ category introduced by Almeida et al. [36]. 82.7% (795/961) of these nearly complete DGs and 88.4% (482/545) of the high-quality MAGs did not affiliate with any described species when using the Genome Taxonomy Database Toolkit (GTDB-Tk) (Fig. S8 in Additional file 2).

In order to determine the total number of species, we also clustered the initial 965 DGs and 1,619 MAGs using a multi-step distance-based approach (95% ANI). This resulted in 1,781 (macroalgae: 1,185) inferred prokaryotic species, 1,689 *Bacteria* (macroalgae: 1,182) and 49 *Archaea* (macroalgae: 3) (Table S3 in Additional file 3). *Archaea* exhibited only low overall abundances, as did *Firmicutes*. The latter, however, were frequently isolated due to cultivation bias (Fig. 5a).

### 15/138 species-level genomes of novel core/dominant phycosphere bacteria

We analyzed all genomes representing core/dominant phycosphere genera, consisting of 28/228 (macroalgae: 25/223) DGs and 282/57 (macroalgae: 263/57) MAGs. These included 15 novel core and 138 novel dominant species. The most frequent core and dominant phycosphere genera comprised *Sulfitobacter*, *Aquimarina*, *Maribacter*, *Tenacibaculum*, *Ruegeria*, *Yoonia*-*Loktanella*, *Erythrobacter*, *Microtrichaceae* unc., *Saprospiraceae* unc. and *Granulosicoccus* (Fig. 3, Table S3 in Additional file 3). Those represented by high numbers of species exhibited similar abundance patterns on all macroalgae and were hardly found in the control samples. At the family level, an even higher number of isolated strains represented core/dominant phycosphere bacteria (Fig. S4 in Additional file 2).

### Phycosphere *Bacteroidota* harbored high proportions of as yet unknown genes

Automatic annotation of DGs and MAGs based on the EggNOG v5, COG (2020) and Pfam (2020) databases resulted in function predictions for on average 80.9%, 75.9% and 77.1% of the genes, respectively (Fig. S9 in Additional file 2). However, when using the more specific UniProtKB and KEGG databases, 46.8% and 75.6% of the genes did not yield any annotations. Among all phyla, the 376 genomes obtained from cultured *Bacteroidota* (305 from macroalgae) had the highest proportion of unknown genes. This exemplifies that macroalgae-colonizing *Bacteroidota* constitute a particularly rich resource of as yet unknown gene functions. Genomes from macroalgal phycosphere bacteria were on average larger than those from sediment and seawater bacteria, with seawater samples featuring the smallest average genome size (Fig. 5b).

It is beyond the scope of this study to interpret the functional potential of all genomes. Instead, we focus on two prevalent traits of phycosphere bacteria, namely their potentials to degrade algal polysaccharides and to synthesize bioactive compounds (Fig. 5b).

### Phycosphere *Bacteroidota* dominated the degradation of algal polysaccharides

We searched all DGs and MAGs for carbohydrate-active enzyme (CAZyme) genes and identified 292,848 homologs. *Bacteroidota* (717)*, Chloroflexi* (70)*, Planctomycetota* (68), *Verrucomicrobiota* (66), *Acidobacteriota* (32) and *Actinobacteriota* (151) genomes encoded the highest proportions of catabolic CAZymes, i.e., glycoside hydrolases (GHs), carbohydrate esterases (CEs), carbohydrate-binding modules (CBMs), auxiliary activities (AAs) and polysaccharide lyases (PLs) (Fig. S10 in Additional file 2). The majority (61.8%) of CAZyme genes were found in *Bacteroidota*, corroborating the pivotal role that members of this phylum play in the degradation of algal polysaccharides [37]. Predicted CAZymes comprised 30.6% GHs, 29.9% glycosyltransferases (GTs), 15.1% CEs, 10.2% CBMs, 5.1% PLs and 5.1% AAs.These proportions were similar across samples (Table S3 in Additional file 3). AAs were more prevalent in macroalgae-associated *Alphaproteobacteria* than in any other phylum (Fig. S10, pie in Additional file 2). Many of the so far described 17 AA families represent lytic polysaccharide monoxygenases, e.g., AA9 acts mainly on cellulose and xyloglucan, AA11 on chitin, AA13 on starch and AA14 on xylan. This suggests a distinct role of *Alphaproteobacteria* in algal polysaccharide degradation.

More than 40% (121,015) of the CAZymes featured signal peptide predictions. Few signal peptides were predicted for GTs (2.4%) and AAs (1.7%), whereas much higher proportions were predicted for PLs (76.5%), GHs (55.6%) and CEs (42.9%), indicating periplasmic or extracellular locations (Table S3 in Additional file 3). These proportions were similar across samples. Surprisingly, the proportion of predicted secreted sulfatases, required for desulfation of sulfated algal polysaccharides [38], were ~ 11% and ~ 13% higher in seawater and sediments than in phycosphere bacteria (Table S3 in Additional file 3). In particular, *Planctomycetota* and *Verrucomicrobiota* featured high numbers of CAZyme and sulfatase genes (Fig. S11 in Additional file 2).

We classified candidate loci for polysaccharide degradation into four categories (Fig. S12a in Additional file 2): (i) PULs consisting of CAZyme genes and *susCD* pairs, (ii) PUL-like clusters with CAZyme genes and an encoded TonB-dependent receptor, (iii) CAZyme-rich gene clusters (CGC) consisting solely of CAZymes, and (iv) *susCD* loci without detectable CAZymes. We identified 4,451 PULs, 6,376 PUL-like loci, 19,826 CGCs and 1,699 *susCD* only loci (Table S3 in Additional file 3). The majority were found in DGs (3,461, 3,875, 9,572 and 1,076) (Fig. S13 in Additional file 2, Table S4 in Additional file 3) due to higher overall completeness compared to MAGs. Sulfatase genes were present in 22.3% of the PULs, 5.5% of PUL-like gene clusters, 7.0% of CGCs and 2.9% of *susCD* only loci, underscoring the relevance of polysaccharide sulfation in marine algal polysaccharides (Table S3 in Additional file 3).

**Fig. 6:**
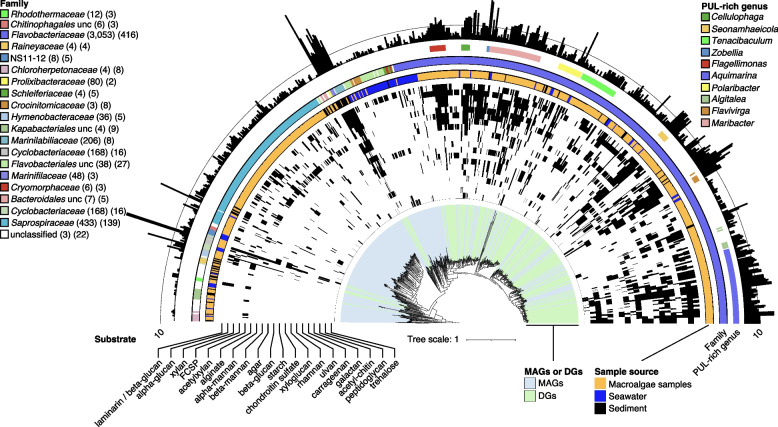
PUL distribution in metagenome-assembled genomes (MAGs) and draft genomes (DGs). Depicted is a phylogenomic tree for all 741 bacterial MAGs (including 27 unclassified MAGs at the root) and DGs based on protein sequences of 43 universal single-copy genes with circles representing (inside to outside): (i) MAGs or DGs, (ii) predicted polysaccharide degradation capacities based on PUL-associated CAZyme annotations, (iii) sample source, (iv) GTDB family classification, v) highlighting of PUL-rich taxa, (vi) bar chart representing the number of predicted PULs. Numbers in parentheses indicate PUL numbers and genome numbers in the corresponding families, respectively

Hierarchical clustering according to Bernard [39] with a 100% distance threshold separated the 4,451 PULs into 2,260 clusters. About one-third (763) contained at least two identical PULs, whereas two-thirds were unique. Few PULs were frequent, as only 1.8% (40) of the clusters had more than ten identical instances. Genomes from macroalgae and sediments contained on average more PULs than those from seawater. Compared to seawater, PUL numbers were 1.6 times higher in phycosphere and 2.8 times higher in sediment genomes (Table S5 in Additional file 3). In particular *Bacteroidota* from the phycospheres (*Flavobacteriaceae*) and sediment (*Marinilabiliaceae*) featured more species than seawater samples and higher numbers of more diverse PULs (Fig. 6). In phycospheres, PUL-rich species mainly belonged to *Zobellia*, *Polaribacter*, *Aquimarina*, *Tenacibaculum*, *Algitalea* and *Maribacter*, representing either core or dominant phycosphere genera (Fig. 6). Additional PUL-rich genera comprised *Cellulophaga*, *Flagellimonas*, *Flavivirga* and *Seonamhaeicola,* which were mainly isolated from macroalgae (Fig. 6). In sediments, *Prolixibacteraceae* and *Marinilabiliaceae* were particularly PUL-rich (both up to 30 PULs), and in seawater *Maribacter* species (up to 24 PULs) [29] (Fig. 6, Table S3 in Additional file 3).

**Fig. 7:**
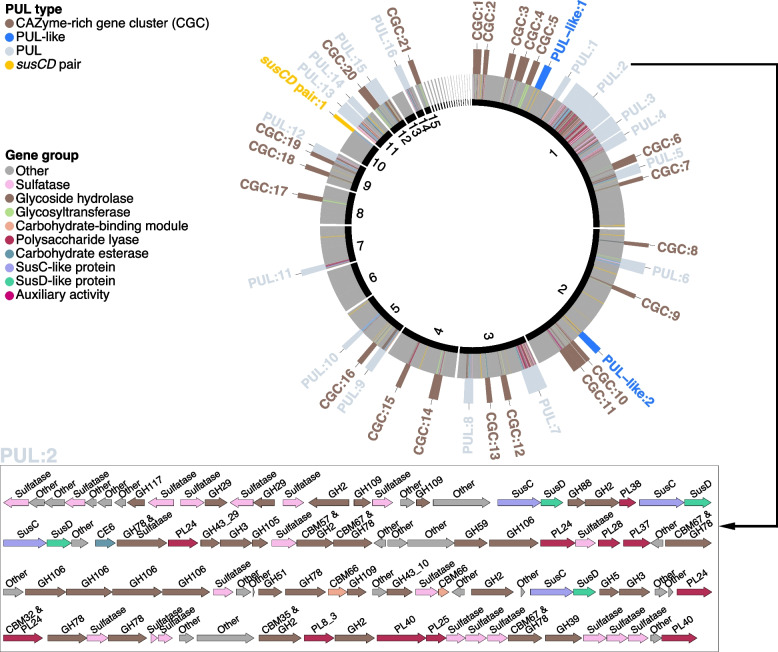
Overview of the *Algibacter* sp. strain 4–1052 draft genome. From inside to outside: (i) contig ID (sorted by lengths), (ii) CAZyme and sulfatase genes, (iii) positions of loci potentially involved in polysaccharide degradation, (iv) locus type. Inset: Structure of the longest PUL (PUL:2)

The largest PUL (tandem repeat and hybrid *susCD* PUL) of in total 99 genes (48 CAZyme genes) was found in the core phycosphere species *Algibacter* sp. 4-1052 (*Bacteroidota*; *Flavobacteriaceae*) isolated from *Ulva* sp. (Table S4 in Additional file 3). This PUL, rich in GH29, GH106, PL40, PL25 and sulfatase genes*,* may target fucoidan, ulvan and/or rhamnogalacturonan (Fig. 7). The largest CGC (93 genes) was found in a *Gaetbulibacter* species (*Bacteroidota*; *Flavobacteriaceae*) isolated from *Grateloupia* sp. and sediment (Table S4 in Additional file 3). *Draconibacterium* sp. X8 (*Bacteroidota*; *Prolixibacteraceae*) isolated from *Gelidium* sp. featured the highest number of PULs (50) (Table S3 in Additional file 3), the third highest number of CAZyme genes (412), and the highest percentage of CAZymes in PULs (85.7%).

Sequence analysis of PUL-encoded SusC and SusD substrate-binding and take-up proteins can provide hints on possible glycan substrates [40]. Hence, we combined phylogenetic SusC/D protein tree and PUL CAZyme composition analyses to infer possible substrate classes (Additional file 1). The complete SusC/D protein tree featured 157 SusD and 159 SusC clusters. Each cluster contained at least five SusC/D protein sequences and represented PULs of similar CAZymes composition (Fig. S14 in Additional file 2, Table S5 in Additional file 3). Examples are GH3/GH16 for β-glucans (including laminarin), GH13/GH65 for α-glucans or PL6/PL7/PL12/PL17 for alginate. The most frequent predicted substrates were xylose-containing polysaccharides (779) (178 PULs containing solely putative acetylxylan esterases of the CE1, CE3 or CE4 families), β-glucans/laminarin (618), α-glucans (482), fucose-containing sulfated polysaccharides (FCSPs) (444), alginates (426), α-mannans (268), β-mannans (220), sulfated α-rhamnose-containing polysaccharides (219), agars (192), chondroitin (158), xyloglucan (133) galactans (128), ulvans (127), starch (114), carrageenans (109), chitin (109), pectin (72), peptidoglycan (69), levans/fructans (36) and porphyran (31) (Fig. S14 in Additional file 2, Table S5 in Additional file 3). In general, a large number of PULs were rich in sulfatase or deacetylase genes, suggesting sulfated and acetylated polysaccharide substrate targets (Table S6 in Additional file 3). Of course, PULs with common substrate predictions were not exactly identical due to the extent of variation in PUL compositions (Table S5 in Additional file 3). Consequently, a wide range of as yet undescribed PULs was identified, and some larger PULs were ascribed to multiple polysaccharide substrates (Fig. S14 in Additional file 2, Table S5 in Additional file 3).

### Phycosphere taxa, in particular *Bacteroidota*, were surprisingly rich in biosynthetic gene clusters

We identified 8,810 putative BGCs (Table S7 in Additional file 3). Predicted product classes comprised terpenes (28.3%), bacteriocins (12.3%), non-ribosomal peptides (NRPS) (10.5%) and NRPS-like clusters (8.0%), homoserine lactones (7.8%), type III polyketide synthases (7.5%), type I polyketide synthases (5.9%) and beta-lactones (5.4%).

Since DGs were generally more complete than MAGs (Fig. S15 in Additional file 2), they featured lower proportions of incomplete BGCs (Fig. S16 in Additional file 2). 20.1% of the 4,816 BGCs predicted in DGs resided on contig edges and were thus potentially incomplete, while this was the case for 73.2% of the 3,994 BGCs predicted in MAGs. We observed clear distinctions between phyla (Fig. S17a in Additional file 2), but no clear trends were observed for BGC families with respect to habitat (Fig. S17b in Additional file 2). Still, we identified more than 483 BGCs > 50 kbp and 1,561 BGCs > 30 kbp (Table S7 in Additional file 3). The largest was identified in a *Streptomyces* species retrieved from *Gelidium* sp. It coded for no less than 22 PKS and NRPS modules.

**Fig. 8:**
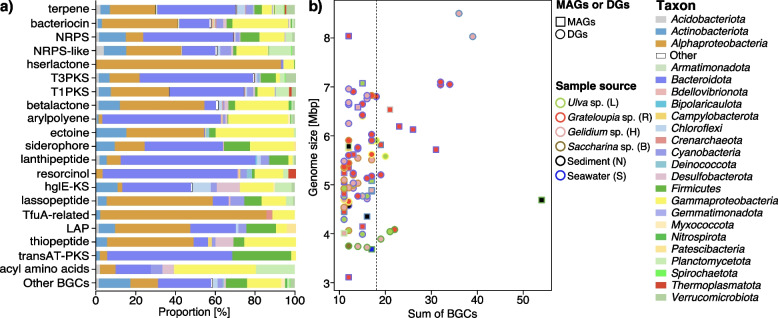
Biosynthetic gene cluster composition and distribution among 1,619 metagenome-assembled genomes (MAGs) and 965 draft genomes (DGs) from all samples. **a** Proportions of BGC types in MAGs and DGs of different phyla. **b** Top 100 BGCs versus genome sizes with MAGs represented by squares and DGs by circles. Fill colors represent taxonomies, and border colors sample sources. Circle and square sizes correspond to genome sizes. The right side of the dotted line represents the top 20 with the largest number of BGCs, which mainly belong to the *Bacteroidota*. Details are provided in Table S4 in Additional file 3

Ninety-three of the top 100 genomes with the highest number of BGCs belonged to phycosphere bacteria and ten of the top 20 genomes with the highest number of BGCs belonged to phycosphere *Bacteroidota* (Fig. 8b). The latter indicates that the potential for secondary metabolite production in this phylum may as yet have been underestimated. *Bacteroidota* had high proportions of BGCs for terpene and NRPS biosynthesis (Fig. 8a), e.g., the novel core phycosphere species *Aquimarina* sp. 2-328 (Table S7 in Additional file 3).

**Fig. 9:**
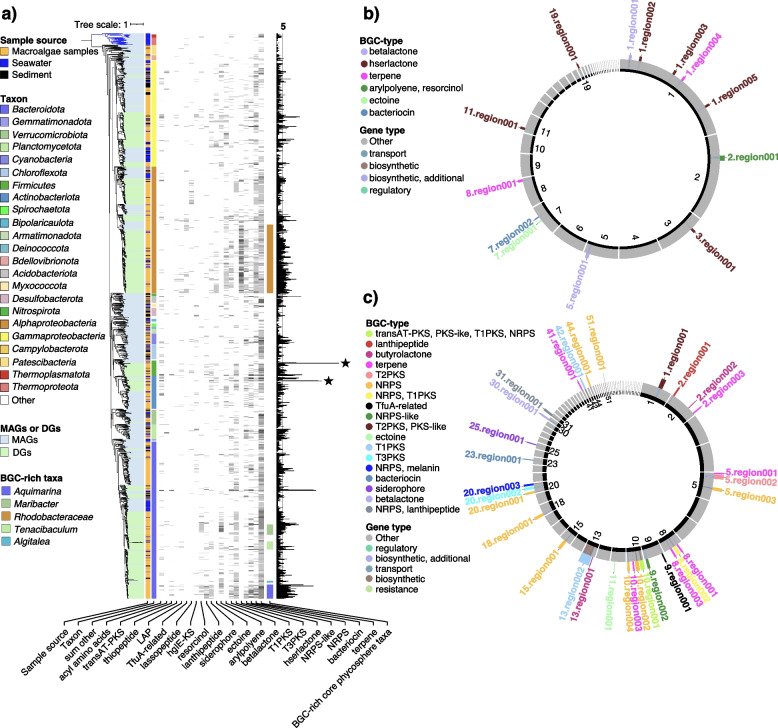
Overview of biosynthetic gene clusters. **a** Phylogenomic tree for all 2,584 bacterial metagenome-assembled genomes (MAGs) and draft genomes (DGs) based on protein sequences of 43 universal single-copy genes (blue branches represent *Archaea*). From left to right: (i) origin: MAG or DG, (ii) sample source, (iii) GTDB phylum annotation, (iii) the number of various abundant BGCs, (iv) BGC-rich core phycosphere taxa, and (v) the sum of BGCs. The two strains with the most BGCs *Ruminiclostridium* sp. (*Firmicutes*) and *Streptomyces* sp*.* (*Actinobacteriota*) are marked by asterisks. **b** Overview of BCGs in *Roseovarius* sp. strain 2–342. From inside to outside: (i) contig ID (sorted by lengths), (ii) genes related to BGCs, (iii) BGC type, (iv) BGC identifier. **c** Overview of BCGs in *Streptomyces* sp. strain 3–371. From inside to outside: (i) contig ID (sorted by lengths), (ii) genes related to BGCs, (iii) BGC type, (iv) BGC identifier

Most BGCs were identified in *Bacteroidota*, *Alphaproteobaceria*, *Gammaproteobacteria*, *Firmicutes* and *Actinobacteriota* (Figs. 9a, S16 in Additional file 2), all taxa that are rich in core phycosphere bacteria. *Firmicutes* and *Actinobacteriota* are known for abundant secondary metabolite production [33]. We found 559 BGCs in 151 *Actinobacteriota* genomes (including 100 MAGs), covering a broad diversity of predicted products. While the highest number of BGCs (54) was found in a *Firmicutes* MAG from sediment (Fig. 8b), the second (39) and third (36) highest numbers were found in draft genomes of actinobacterial *Streptomyces* strains 3-371 isolated from macroalgae (Fig. 9c). *Alphaproteobacteria* were particularly rich in BGCs, many coding for homoserine lactones, especially the core phycosphere family *Rhodobacteriaceae* (Fig. 9a, b), e.g., the phycosphere species *Roseovarius* sp. 3-342 (*Rhodobacteraceae*) isolated from *Gelidium* sp (Fig. 9b, Table S7 in Additional file 3) contained six related gene clusters.

## Discussion

Approximately 40–80% of the *Bacteria* and *Archaea* on Earth reside in biofilms [41]. Selected biofilms have been extensively studied [33], but little is known about the diversities and functions of marine macroalgal biofilms, in particular on a global scale. Algal colonization is influenced by stochastic as well as deterministic processes. While functionally redundant yet taxonomically distinct species can replace each other (stochastics) [4, 22], it has also been shown that phycosphere bacteria share a robust pool of essential genetic functions (determinism) [23]. Both allow for largely varying phycosphere compositions, but more selective processes must be at play, since it has also been reported that phycosphere communities are at least in parts host-specific [21].

We observed surprisingly stable core phycosphere compositions across all four studied algae species on genus and family levels, in particular with respect to dominating members of *Alphaproteobacteria*, *Gammaproteobacteria* and *Bacteroidota*. Core genera, while representing only a minor proportion of the phycosphere diversities, made up a major proportion of the phycosphere abundances, even though their relative proportions fluctuated throughout seasons. This is unlikely a purely biogeographic effect of sampling in close proximity, because some core genera have also been described in other studies [22, 23]. Phycospheres of *Ulva australis* for example feature high abundances of *Lewinella* (*Lewinellaceae*), *Maribacter* (*Flavobacteriaceae*), *Loktanella *(*Roseobacteraceae*), *Sulfitobacter* (*Roseobacteraceae*) and *Erythrobacter* (*Erythrobacteraceae*) [4]. Also, *Granulosicoccus* has been shown to dwell on multiple macroalgal species [10].

It seems that the sampled reef harbors a pool of common and widespread potential phycosphere bacteria, some of which are more successful in macroalgal colonization than others, in particular members of the core/dominant genera. Superimposed are host-specific and stochastic phycosphere taxa. To elucidate, whether or not the core/dominant community is stable over longer periods of time, or gradually changing as it is part of a larger pool of suitable bacteria that can functionally replace each other, would require multiple years of consecutive studies and thus remains an open question.

The *Flavobacteriaceae* and *Saprospiraceae* core families are of particular interest. *Flavobacteriaceae* are known to degrade biopolymers [40] and have been found in various marine [42] and terrestrial habits [42, 43], and in association with microalgae [40], macroalgae [3, 4] and marine animals [42]. Symbiotic *Flavobacteriaceae* are also known to produce vital compounds for their hosts [44, 45]. For instance, members of the genus *Zobellia* are known to induce morphogenesis of *Monostroma oxyspermum* green algae [45]. Likewise, *Saprospiraceae* have been isolated from diverse marine habitats, including seawater, particles, sediments and macroalgae such as *Ulva* spp. and *Delisea pulchra* [3, 4, 46]. Members of the *Saprospiraceae* are likely involved in the breakdown of complex organic compounds [47] and in algal endosymbiosis [43].

*Verrucomicrobiota* are also known to be associated with macroalgae [3]. Members of the *Verrucomicrobiota* and its sister phylum *Planctomycetota* [48] have been suggested as specialists for sulfated algal polysaccharides, since their genomes tend to feature copious sulfatase genes [49]. Verrucomicrobial *Rubritaleaceae* are known to feature biofilm-forming bacteria [50] and were abundantly present on *Saccharina* sp. winter samples. The latter might be a consequence of *Saccharina* sp. being in the seeding stage during this time. Recent studies indicate that some free-living *Verrucomicrobiota* specialize in the degradation of fucose- and rhamnose-rich algal polysaccharides including fucoidan [49, 51].

### Expanding the catalog of known algal phycosphere bacterial species

Most bacteria from marine macroalgae resist common cultivation techniques, and those that have been cultured mostly belong to the ‘rare biosphere’ [52]. In this study, we could culture strains from 367 genera (macroalgae: 302), including six (*Hellea*, *Algitalea*, *Sulfitobacter*, *Granulosicoccus*, *Leucothrix*, *Robiginitomaculum*) core and ten (*Maribacter*, *Tenacibaculum*, *Aquimarina*, *Erythrobacter*, *Planktotalea*, *Yoonia*-*Loktanella*, *Ruegeria*, *Acinetobacter*, *Pseudahrensia*, *Celeribacter*) dominant phycosphere genera (Fig. 3) (Table S2 in Additional file 3). The cultured core phycosphere species mainly belong to the *Rhodobacteraceae* and *Flavobacteriaceae* families (55.4% of the total). In addition, 29 strains were obtained with either unresolved or incomplete taxonomies. About eight to nine times as many dominant than core species were obtained using cultivation. Conversely, four to five times as many MAGs of core than dominant species were obtained using metagenomics. This illustrates that some core taxa are difficult to cultivate and that a large fraction of the core phycosphere species remains without a cultured representative. However, as exemplified by our study, macroalgal phycospheres also host high numbers of cultivable species that can be readily explored.

As of June 2022, the number of validly published prokaryote species stood at 18,297 with a total of 3,365 genera (names validly published under the ICNP, w/o synonyms; https://lpsn.dsmz.de/text/numbers). These numbers are far from reflecting the existing natural bacterial diversity. Among the so far validly described cultured species, only 203 were obtained from macroalgae. In this study, we isolated 689 novel species, the most prevalent of which need to be validly described. Still, much of the diversity of the macroalgal microbiome remains uncultured, including prevalent clades with important ecophysiological functions.

### Polysaccharides and PULs

Variations in chemical structures of macroalgal polysaccharides depend not only on the species, but also on the body parts and developmental stage of the sampled macroalgae, season, and other environmental factors [25]. Bacteria that degrade such polysaccharides require numerous or adaptive, complex PULs to account for these variations. A single PUL often encodes the entire apparatus to degrade a specific glycan, but in the case of chemically complex glycans, it has been shown that multiple PULs can be involved [53]. This might explain, why in *Bacteroidota* we observed not only large numbers of PULs, but also a high diversity of CAZyme genes, in particular in large hybrid *susCD* PULs (Fig. 7).

The current challenge is not to obtain more PUL data, but rather to infer the functions of the plethora of PULs that have already been identified. The PUL gene repertoire and diversity in phycosphere *Bacteroidota* suggest a high level of functional redundancy, which may enable adaptation to various macroalgal hosts. This redundancy might be the result of PUL acquisitions via horizontal gene transfer [23, 54]. Indicative of the latter is that PUL patterns were not always congruent with the 16S phylogeny (Fig. 6).

We found similar collective PUL repertoires in the epiphytic bacteria of all sampled macroalgae, which supports the presence of functional guilds within the macroalgal microbiome with members that can functionally fill in for each other. In particular, *Bacteroidota* in all sampled habitats were rich in PULs, underpinning the exceptional role that *Bacteroidota* play in marine polysaccharide degradation. PULs predicted to target well-defined, structurally simple polysaccharides, such as laminarin, starch and alginate, comprised fewer CAZyme genes and were more conserved than PULs predicted to target more complex polysaccharides, such as carrageenans and ulvans. Some of the larger, complex PULs might actually address multiple substrates. For example, cluster 27_1 in the SusC/D protein tree comprised carrageenan PULs with a family GH5_2 gene (Table S5 in Additional file 3). The latter might target either xylan (endo-β-1,4-xylanase function) or cellulose (endo-β-1,4-glucanase function), which often coexist with carrageenan in natural habitats. Likewise, PULs predicted to target ulvans and rhamnogalacturonans contained additional endohydrolases seemingly unrelated to the actual substrate. The reason might be that algal sulfated polysaccharides are rarely homogeneous, but mostly complex heterogeneous mixtures of different glycans [55]. Further predicted substrates included sulfated α-rhamnose- and α-galactose-containing polysaccharides, FCSPs, agars, and fructose-rich polysaccharides such as fructans and levans, plus bacterial polysaccharides such as gellan, peptidoglycan, O-antigenic side chains, eukaryotic N-glycans, and common small sugar molecules, such as trehalose and sialic acids (Additional file 1).

Recalcitrant macroalgal polysaccharides eventually end up in the sediment [1], and some sediment taxa with high numbers of CAZymes and PULs, such as bacteroidotal *Marinilabiliaceae* and *Prolixibacteraceae*, have the potential to further degrade such polysaccharides (Figs. 6, S18 in Additional file 2). In our samples, *Marinilabiliaceae* from sediments featured similar PUL numbers than macroalgal core taxa (Fig. 6). We therefore suppose that *Marinilabiliaceae* play an important role in the degradation of macroalgal polysaccharides in marine sediments. *Planctomycetota* and *Verrucomicrobiota* also seem to play such a role in sediments, as they featured more CAZyme genes than those from macroalgal samples, but fewer sulfatases (Fig. S11 in Additional file 2). Interestingly, *Planctomycetota* and *Verrucomicrobiota* in seawater featured more sulfatase genes than those from macroalgae and sediments. This is likely a consequence of different dominating taxa (Fig. S11 in Additional file 2), and might indicate that those in phycospheres seem to preferentially degrade less sulfated and thus more accessible polysaccharides.

### Secondary metabolites

Phycosphere bacteria are known to produce secondary metabolites, including antibacterial substances [46, 56]. The latter are crucial for maintaining a specific phycosphere community composition [57].

Phycosphere bacteria in our samples had larger genomes and relatively more BGCs compared to seawater and sediment bacteria (Fig. 5b). There were also notable taxonomic differences (Figs. 3, S4 in Additional file 2). *Flavobacteriaceae* and *Rhodobacteraceae* comprised core/dominant phycosphere taxa with remarkably high BGC proportions (Fig. 9a), for example, members of the genera *Maribacter*, *Algitalea*, *Tenacibaculum*, *Aquimarina*, *Ruegeria* and *Sulfitobacter* (Fig. 9a). Six of the topmost ten abundant phycosphere genomes originated from these two families, which is why respective isolates should be prime targets for the discovery of novel bioactive agents. The *Actinobacteriota* constitute a prime source for the discovery of new drugs. In particular, *Streptomyces* species are prolific producers of antibiotics and other natural agents (Fig. 9a, c) [58]. Due to the depletion of secondary metabolite resources of terrestrial actinomycetes, representatives from marine macroalgal phycospheres, such as *Streptomyces* spp., may become future viable substitutes. For example, actinobacterial *Microtrichaceae* in this study represented a core phycosphere family. While we did not succeed in cultivating a representative species (but did obtain 62 MAGs), macroalgal phycospheres are rich in *Microtrichaceae* and thus a viable resource for the isolation of novel marine actinomycetes (Fig. S4 in Additional file 2). Further non-core/dominant phycosphere genera with members rich in BGCs comprised *Kordiimonas*, *Shewanella*, *Kocuria* and *Bacillus*.

Homoserine lactones, such as N-acyl-L-homoserine lactones (AHLs), act as messenger molecules that enable bacteria to collectively change gene expression, a process known as quorum sensing (QS) [59]. Bacteria isolated from plants [59], macroalgae [60] and animals [61] have been shown to produce AHLs. The first marine phycosphere bacterium for which QS was shown was isolated from the red macroalga *Delisea pulchra,* which appears to have developed natural defense mechanisms to prevent microbial surface fouling [60]. Likewise, almost 40% of the strains isolated from the brown macroalga *Fucus vesiculosus* were able to degrade AHLs [62], suggesting that inhibition of QS could be widespread among algae-associated bacteria. A total of 690 homoserine lactone BGCs were predicted in our study, most in *Rhodobacteraceae*, representing one of the most prevalent core phycosphere families (Figs. S4 in Additional file 2, 9a). *Rhodobacteraceae* could thus play a key role in controlling algae colonization [59].

The bacterial endosymbiont *Cd.* Endobryopsis kahalalidefaciens of *Bryopsis* sp. green algae has abundant and diverse NRP-synthesis BGCs that it uses to produce toxins for the defense of its host [44]. Pure cultures of symbiotic bacteria are usually hard to obtain, whereas epiphytic *Bacteroidota* of macroalgae also have rich NRPS-synthesizing BGCs and are more readily available (Figs. 6a and 9a). Still, the successful translation of NRPS BGCs from phycosphere bacteria via NRPS/PKS megasynthases for drug discovery remains a major challenge for the future.

Terpenes constitute another diverse class of compounds that are mainly produced by plants and fungi [63]. Also *Cyanobacteria* [32] and *Planctomycetota* [48] are known to feature terpenoid biosynthesis pathways. Both are well represented among the dominant phycosphere taxa, suggesting the production of terpenoid compounds. In addition, we observed the presence of terpene synthesis gene clusters in *Alphaproteobacteria* and in *Bacteroidota* (Figs. 6a and 9a). Most of the predicted BGC products were unclassified (Table S7 in Additional file 3), which reflects our limited knowledge on secondary metabolites and substantiates that phycosphere bacteria represent a rich resource of as yet unexplored biosynthetic functions.

## Conclusions

To our knowledge, this dataset represents the largest effort so far on phycosphere bacteria in terms of phylogenetic coverage, cultured isolates and genome data. Our study not only corroborated that all sampled macroalgae were characterized by similar phycosphere communities, but also yielded 689 isolates of novel species. In particular, we succeeded in cultivating a sizable number of strains of core and dominant phycosphere members for future in-depth functional studies. At the same time, we expect that the genome data provided in this study will act as a valuable search space for future metatranscriptome studies of entire macroalgal microbiomes.

As yet, abundant heterotrophic phycosphere bacteria, in particular from the *Planctomycetota*, *Verrucomicrobiota* and *Chloroflexota*, remain uncultured, and thus should be a focus in future studies. Such studies should also include more algal species and multiple sites. Our data represents a stepping stone in this direction and will hopefully serve as a sound basis for further and refined research on the specific adaptations of core phycosphere bacteria.

## Materials and methods

### Sampling

We sampled a coastal area in Weihai, China (122.12 N, 37.56 E) in 2018/19 on October 15^th^, January 15^th^, May 1^st^, and August 1^st^. Live *Ulva* sp. (green algae), *Saccharina* sp. (brown algae), *Grateloupia* sp. (red algae), *Gelidium* sp. (red algae), surrounding seawater (-0.1 to -0.5 m) and surface sediment (~ 5 m depth) were collected in triplicates in sterile plastic bags, kept on ice and transported to the laboratory within 2 h. At each time point, all four macroalgal species were sampled, with the exception of August, where *Saccharina* sp. was decomposed due to summer temperatures. In total, we sequenced 23 metagenomes and 92 16S rRNA gene tag libraries, and isolated 5,527 bacterial strains, 965 of which were draft sequenced (Fig. 1).

### Cultured bacteria

Extraction and isolation by dilution of bacteria from phycosphere, seawater and sediment samples are described in Additional file 1. Two media were used for plating, modified 2216E and modified VY/2 medium (Additional file 1). Colonies were selected depending on color, size, and shape. Picked colonies were purified by serial cultivation on plates with identical media. Purified strains were stored at -80 °C in sterile 1% (w/v) saline medium with 15% (v/v) glycerol.

For 16S rRNA gene sequencing the universal bacterial primers 27F and 1492R were used as described elsewhere [64]. PCR products were subsequently Sanger-sequenced by BGI Co. Ltd. (Qingdao, China). Resulting sequences were classified using the EzTaxon server [65] to identify known taxa (≥ 98.7% similarity to published type strains). Additional taxonomic assignments were done using SILVA v138.1 [66].

Strains of novel species lacking reference genomes in the Type Strains Genome Database [67] and strains present on all macroalgal samples were selected for sequencing. Sequencing was performed by Beijing Novogene Biotechnology (Beijing, China) on a NovaSeq (Illumina, San Diego, CA, USA) with 150 bp PE reads at ≥ 100 × coverage. Reads were quality-filtered and assembled with SPAdes v3.9.1 [68] (–careful –cov-cutoff) with k-mer sizes from 27 to 127 bp and a minimum scaffold length of 200 bp. Further details are provided in Additional file 1.

### Environmental 16S rRNA gene tags

We sequenced 16S rRNA gene V3-V4 regions using primers 341F and 806R as described elsewhere [69]. Sequencing was carried out on the Illumina NovaSeq platform using 2 × 250 bp chemistry at Guangdong Magigene Biotechnology Co., Ltd. (Shanghai, China). Cutadapt v3.0 [70] was used to remove primers and adapters. Reads were trimmed to ≥ Q25, and dereplicated using DADA2 [71] (paired-end setting) resulting in tabulated read counts of amplicon sequence variants (ASVs). ASV taxonomies were assigned based on a ≥ 97% similarity criterion to 16S rRNA sequences in the SILVA v138.1 database, and a 97% similarity threshold was also used for creating OTUs in SILVAngs [72]. Chloroplast and mitochondria sequences were removed from subsequent analyses.

### Metagenome-assembled genomes (MAGs)

Library construction and sequencing of metagenomes were performed as presented in Additional file 1. A total of 1.4 Tbp (avg. 65 Gbp per metagenome) were generated (Table S1). Read quality filtering was done with BBDuk v35.14 (http://bbtools.jgi.doe.gov) and verified with FastQC v0.11. Reads from each sample were subsequently assembled individually using MEGAHIT v1.2.9 [73] with a minimum scaffold length of 2.5 kbp.

BAM files were generated for each metagenome by mapping reads onto assemblies with BBMap v38.86 (minid = 0.99, idfilter = 0.97, fast = t and nodisk = t.) Initial binning was performed from within anvi’o v6.2 [74] using CONCOCT v0.4.0 [75], MaxBin v2.1.1 [76] and MetaBAT v0.2 [77]. Resulting bins were combined with DAS Tool v1.1 [78] in order to find an optimal set. Anvi’o was used for manual bin refinement and CheckM [79] and Prokka v1.13 [80] were used for estimating completeness of MAGs. Genomes were classified into high-, medium-, and low-quality classes according to MIMAG criteria [35].

MAGs were denoted by an initial capital letter specifying the sample (B = *Saccharina*, L = *Ulva*, H = *Grateloupia*, R = *Gelidium*, S = seawater, N = sediment), followed by a number representing the season (1 = autumn, 2 = winter, 3 = spring, 4 = summer), followed by the binning program, and a terminal numeric identifier (Table S3).

### Taxonomic inference of MAGs and draft genomes

Initial taxonomic classification of MAGs and draft genomes was done with GTDB-Tk v1.3.0 [81] using the default classify_wf command. In addition, 16S rRNA genes were predicted with Barrnap (https://github.com/tseemann/barrnap) and classified with SILVA v138.1. Inconsistent classifications were resolved by majority rule. For MAGs without 16S rRNA gene, the SILVA taxonomy was taken when both SILVA and GTDB predictions agreed (Fig. S8).

### Diversity and core taxa analyses

The methods used for α- and β-diversity analyses are described in Additional file 1. Only genera and families were included that were present in ≥ 85% of a given set of analyzed samples and accounted for ≥ 1% of sequences in at least one sample. For macroalgae, these taxa were categorized as follows: (1) core phycosphere taxa (present on all four macroalgal species), (2) dominant phycosphere taxa (present on three macroalgal species), and (3) host-specific phycosphere taxa (present on one or two macroalgal species). Seawater and sediment core taxa were computed correspondingly.

### Phylogenetic analyses and OTU-clustering of MAGs and draft genomes

Phylogenomic analyses of MAGs and draft genomes were executed within anvi’o v6.2 based on concatenated ribosomal protein sequences (Additional file 1). Maximum-likelihood trees were constructed in FastTree v2.1.5 [82] (default settings) and visualized in iTOL v6.5.6 [83]. Draft genome and MAG dereplication were performed using dRep v3.2.0 [84] based on a > 65% alignment and a genome-wide ANI threshold of 95% (-nc 0.65, -sa 0.95). The dRep program was also used to compare these draft genomes to 14,131 published species reference genomes from the GCM [34] and public database (https://www.ncbi.nlm.nih.gov/). Draft genomes exhibiting an ANI < 0.95 were designated as different species.

### Functional annotations

Genes were predicted using Prodigal v2.6.3 [85] and annotated with Prokka. Additional annotations were performed using Diamond v0.9.24.125 [86] searches in ‘verysensitive’ mode against the UniRef100 [87] (as of September 2020) and COG [88] databases, as well as HMMER v3.1b2 [89] searches against the Pfam [90] database (as of September 2020). Further annotations were done by aligning genes to the EggNOG 5.0 [91] database using eggNOG-mapper v2.0.1 [92], peptidases were annotated using BLASTp searches against the MEROPS v9.13 database [93]. Biosynthetic gene clusters were identified using antiSMASH v5.0 [94] with default parameters. Signal peptides were predicted using SignalP v5.0 [95].

### Prediction and annotation of PULs and CAZymes-rich gene clusters

Genes coding for carbohydrate-active enzymes (CAZymes) were annotated as described in Krüger et al. [37] using a combination of HMMER searches against the dbCAN v2.0.11 [96] database in conjunction with Diamond v0.9.24.125 searches against the CAZy database [97] as of July 2020. Genes coding for sulfatases, SusC- and SusD-like proteins were predicted using corresponding HMMER and TIGRFAM profiles (Additional file 1). PULs and other CAZyme-rich gene clusters were predicted as described in Francis et al. [98] with a sliding window of ten genes. In addition, we used dbCAN2 [96] to identify such clusters.

### Protein phylogenies

Amino acid sequences were aligned using MAFFT v7.313 [99] with L-INS-I and curated manually. RaxML [100] was used to select the best fitting amino acid substitution model, which was subsequently used to generate maximum likelihood trees in FastTree v2.1.5 with default settings. Trees were visualized using iTOL v6.5.6.

## Supplementary Information

**Additional file 1.** Compilation of supplementary results, supplementary methods and of software tools used in this study.

**Additional file 2:** Compilation of supplementary figures. **Figure S1.** a) Diversities of macroalgae, seawater and sediment samples as assessed by Shannon and Simpson indices as well as Good’s coverage of 16S rRNA ASVs. Statistical significance was assessed using a pairwise Wilcoxon test with Holm *p*-value adjustment for multiple comparisons (*, *p* <  0.05; **, *p* <  0.01; ***, *p* <  0.001). b) Rarefaction curves of the top 200 ASVs for all six samples and all four seasons. **Figure S2.** The most abundant taxa as assessed by 16S rRNA gene amplicon data. **Figure S3.** Phycosphere composition as assessed by 16S rRNA gene amplicon data as a function of host species and season. **Figure S4.** Phylogenies and abundances of the 86 most abundant families as assessed by 16S rRNA gene amplicon sequencing. **Figure S5.** 16S rRNA phylogenetic tree reconstruction for 202 genera that were represented by at least three cultured strains. **Figure S6.** Compositional differences of strains depending on sample source and season. **Figure S7.** Numbers of colony forming units (CFUs) per gram of sample depending on habitat and season. **Figure S8.** Workflow for translating GTDB taxonomic classifications to SILVA taxonomic classifications. **Figure S9.** Proportions of genes within 965 metagenome-assembled genomes (MAGs) and 1,618 draft genomes (DGs) with EggNOG, COG (2020), Pfam, UniProtKB, and KEGG annotations, as well as the percentage of genes lacking any functional annotation. **Figure S10.** CAZymes in metagenome-assembled genomes (MAGs) and draft genomes (DGs) of different phyla. **Figure S11.** CAZymes versus sulfatase gene frequencies in prominent phyla and families as assessed in 1,294 metagenome-assembled genomes (MAGs) and 963 draft genomes (DGs) from all six sample sources. **Figure S12.** Categories of loci used to find putative PULs in this study. **Figure S13.** Histograms of the lengths of the four loci described in Fig. S12 in metagenome-assembled genomes (MAGs) and draft genomes (DGs). **Figure S14.** Tree of all 159 clusters derived from 3,769 PUL-associated SusC-like protein sequences from *Bacteroidota* metagenome-assembled genomes (MAGs) and draft genomes (DGs). **Figure S15.** Basic quality metrics of the 1,619 metagenome-assembled genomes (MAGs) and 965 draft genomes (DGs). Box-plots (A-E) show the minimum value, first quartile, median, third quartile and maximum value. **Figure S16.** Biosynthetic gene cluster (BGC) sizes in genomes from distinct phyla. **Figure S17.** Clustering of biosynthetic gene clusters (BGCs) according to sample type and phylogeny. **Figure S18.** Sizes of PULs and PUL-like loci in genomes from distinct *Bacteroidota* families (categories: hybrid *susCD*, single *susCD*, tandem repeat *susCD*, and tandem repeat plus hybrid *susCD* PULs).

**Additional file 3:** Description of supplementary tables. **Table S1.** Data associated with the 16S rRNA gene amplicon-based community profiling for all six sample sources analyzed in this study. Sequencing, assembly and binning statistics of the 23 metagenome datasets used in this study. These data include the time, season, geographical location, sample, environmental metadata for each sample and library information related to the amplicon sequencing. Furthermore included are summary analyses of the average relative abundances grouped by season and sample type at the genus and family levels, as well as statistical analyses of the proportions of core and dominant taxa in each sample. In addition, this file contains diversity indices, average relative abundances of domain, phylum, family, genus, OTU and ASV levels. **Table S2.** Data associated with the 16S rRNA gene-based community analyses of cultured bacterial strains, including information on sampling time, season, geographical location, source, culture conditions, 16S rRNA sequence information, new species attributes and taxonomic status information. Included are also summary analyses about average relative abundances at phylum, family, genus and OTU levels, as well as core taxa analyses results at the family and genus levels (matched to the 16S amplicon data). In addition, the file contains EZcloud and SILVA 138 sequence alignment results. **Table S3.** Summary data on the 1,619 MAGs and 965 draft genomes, including completeness, contamination, contig number, tRNA number, quality classification, size (Mbp), N50 value, species cluster ID in dRep, and the annotation results from GTDB SR202, EZcloud and SILVA 138 ordered according to their positions on the phylogenetic tree in Fig. 4. **Table S4.** Summary information about the four categories of PULs / PUL-like loci used in this study that were found with sliding window lengths from 1 and 10. The information includes: taxonomic affiliation, length (number of genes), number and type of comprised CAZyme genes, PUL composition (CAZyme genes, *tonB*, *susCD*, sulfatase genes), information on *susCD* genes in classical PULs and the density of CAZyme genes in each PUL. **Table S5.** Information on PULs from this study and published reference PULs, including descriptions of each PUL cluster in the SusC/D protein trees (single *susCD* PULs, hybrid *susCD* PULs, tandem-repeat *susCD* PULs, and tandem-repeat and hybrid *susCD* PULs). Also included is information about the source genome, the source genome type, its taxonomy and habitat as well as PUL ID, cluster number, number of CAZyme genes, composition (CAZymes gene, *susCD*, TonB and sulfatase genes) and genomes, possible substrate. For classical PULs, detailed information of the SusC/D protein tree is provided, including, gene ID, PUL ID, PUL type, PUL composition and potential substrates. **Table S6.** Details on the four categories of PULs and PUL-like loci used in this study in the 1,619 MAGs and 965 draft genomes, including gene composition. gene locus tags and gene annotations from multiple databases (KEGG, CAZy, EggNOG, COG, SignalP, MEROPS and Pfam). **Table S7.** Details on all BGCs predicted in the 1,619 MAGs and 965 draft genomes. This includes overall function predictions and gene function predictions according to KEGG, CAZy, EggNOG, COG, SignalP, MEROPS and Pfam searches. **Table S8.** Annotated putative PUL substrates based on dbCAN-PUL data (dbCAN-PUL is a database of experimentally characterized CAZyme gene clusters and their substrates), and substrate and enzyme cleavage information from the CAZy database (http://www.cazy.org/). These substrates represent automatically derived similarity-based bioinformatic predictions and are thus not as accurate as biochemically characterizations of PUL functions would be.

## Data Availability

Sequences are available from the European Nucleotide Archive under accessions PRJEB51052 (16S rRNA tags, Table S1), PRJEB50838 (metagenomes and MAGs, Tables S1 and S3), and PRJEB57783 (genomes of cultured bacteria). All deposited strains (Table S2) are available from the Marine Culture Collection of China (MCCC) on request. The presented datasets except for metagenomes are also archived at Zenodo (https://doi.org/10.5281/zenodo.7556438).
