# Supplementary material for: Epiphytic common core bacteria in the microbiomes of co-located green (Ulva), brown (Saccharina) and red (Grateloupia, Gelidium) macroalgae
Source: Microbiome. 2023 Jun 1;11:126. doi: 10.1186/s40168-023-01559-1 (PMC10233909; doi:10.1186/s40168-023-01559-1)
Supplement: Supplementary file 2 — Additional file 1. Compilation of supplementary results, supplementary methods and of software tools used in this study. [file 40168_2023_1559_MOESM1_ESM.docx]

***Microbiome - Additional file 1***

**Epiphytic common core bacteria in the microbiomes of co-located green (*Ulva*), brown (*Saccharina*) and red (*Grateloupia*, *Gelidium*) macroalgae**

De-Chen Lu^1,2,3^, Feng-Qing Wang^2^, Rudolf I. Amann^2^, Hanno Teeling^2*^, Zong-Jun Du^1,3^*

^1^ Marine College, Shandong University, Weihai 264209, China

^2^ Max Planck Institute for Marine Microbiology, Bremen 28359, Germany

^3^ State Key Laboratory of Microbial Technology, Institute of Microbial Technology, Shandong University, Qingdao 266237, China

^*^ Corresponding authors:

Zong-Jun Du, Marine College, Shandong University, No. 180, Wenhua xilu, Weihai, Shandong Province, 264209 P. R. China, e-mail: [duzongjun@sdu.edu.cn](mailto:duzongjun@sdu.edu.cn), phone: +86 0631 5688303

Hanno Teeling, Max Planck Institute for Marine Microbiology, Celsiusstraße 1, 28359 Bremen, e-mail: [hteeling@mpi-bremen.de](mailto:hteeling@mpi-bremen.de), phone: +49 421 2028 976

**Running title:** Microbiomes of marine macroalgae

E-mail addresses and telephone numbers of all authors:

De-Chen Lu 202267000015@sdu.edu.cn +**86 0631 5688303**

Feng-Qing Wang [fwang@mpi-bremen.de](mailto:fwang@mpi-bremen.de) +49 421 2028 9390

Rudolf I. Amann [ramann@mpi-bremen.de](mailto:ramann@mpi-bremen.de) +49 421 2028 9300

Hanno Teeling [hteeling@mpi-bremen.de](mailto:hteeling@mpi-bremen.de) +49 421 2028 9760

Zong-Jun Du [duzongjun@sdu.edu.cn](mailto:%20duzongjun@sdu.edu.cn) **+86 0631 5688303**

**Conflict of interest**

The authors declare no conflict of interest.

**This file includes:**

Supplementary Results

Supplementary Methods

Supplementary Code availability

Supplementary References

**Supplementary results**

*Sequencing and cultivation – basic metrics*

Sequencing of 16S rRNA V3-V4 regions was done for 92 samples: 16x *Ulva* sp. (green algae), 16x *Grateloupia* sp. (red algae), 16x *Gelidium* sp. (red algae), 12x *Saccharina* sp. (brown algae), 15x surrounding seawater (-0.1 to -0.5 m), and 17x surrounding sediment. 10,058,653 amplicons of around 450 bp were obtained (average=109,333, max=282,978, min=24,866, SD=51,366), and clustered into 51,132 ASVs (Table S1 in Additional file 3).

Metagenomes were sequenced of 23 samples: 4x *Ulva* sp., 4x *Grateloupia* sp., 4x *Gelidium* sp., 3x *Saccharina* sp., 4x surrounding seawater, and 4x surrounding sediment. In total 1.3 Tbp of high-quality metagenome data with an average of 14.1 Gbp per sample were generated (Table S1 in Additional file 3). Assemblies ≥2.5 kbp ranged from 0.3 to 0.9 Gbp.

Cultivation of bacteria yielded 5,527 pure cultures (phycospheres: 4,426) representing eight phyla, 444 genera, and 1,235 species (phycospheres: 879), including 52 potential novel genera and 637 potential novel species (Table S2 in Additional file 3). Sequencing of selected strains yielded 965 draft genomes comprising 3.9 Gbp in total (Table S3 in Additional file 3).

*Phycosphere core taxa*

Based on 16S rRNA gene ASVs, *Bacteroidota*, *Actinobacteriota*, *Verrucomicrobia*, *Cyanobacteria*, *Deinococcus*, and *Patescibacteria* relative abundances were notably higher in phycospheres than in surrounding seawater and sediments (Fig. S3 in Additional file 2). *Proteobacteria* and *Actinobacteria* relative abundances were higher in phycospheres and seawater, whereas *Chloroflexi*, *Desulfobacterota* and *Campylobacterota* relative abundances were higher in sediments (Fig. S3 in Additional file 2). We studied seasonal changes in the phycosphere communities of all four macroalgae at the family and genus levels. While seasonal changes were observed, the core community as such was remarkably robust (Fig. 3, S4 in Additional file 2). The following 14 genera from eight families were identified as core phycosphere genera: *Saprospiraceae* unc. (*Saprospiraceae*), *Portibacter* (*Saprospiraceae*), *Lewinella* (*Saprospiraceae*), *Algitalea* (*Flavobacteriaceae*), *Microtrichaceae* unc. (*Microtrichaceae*), Sva0996 marine group (*Microtrichaceae*), *Rubritalea* (*Rubritaleaceae*), *Rhizobiaceae* unc. (*Rhizobiaceae*), *Robiginitomaculum* (*Hyphomonadaceae*), *Hellea* (*Hyphomonadaceae*), *Rhodobacteraceae* unc. (*Rhodobacteraceae*), *Sulfitobacter* (*Rhodobacteraceae*), *Granulosicoccus* (*Granulosicoccaceae*) and *Leucothrix* (*Thiotrichaceae*). All core phycosphere genera combined accounted for more than 70% of the diversity of all macroalgae samples, but only for 20% of the corresponding seawater and sediment samples (Table S1 in Additional file 3, Fig. 3).

*Core taxa - average percentages on all sampled algae*

Core families comprised on average 6.1% (*Gelidium* sp., 29/472), 4.6% (*Grateloupia* sp., 22/478), 3.6% (*Ulva* sp., 17/470) and 5.1% (*Saccharina* sp., 23/452) of all families (Table S1 in Additional file 3). At the same time, they accounted for 89.1% (*Gelidium sp.*, ± 9.3), 85.2% (*Grateloupia* sp., ± 7.5), 88.0% (*Ulva* sp., ± 6.0) and 85.9% (*Saccharina* sp., ± 15.3) of the phycosphere bacterial relative abundances. Core genera comprised, on average, 4.3% (*Gelidium* sp., 42/972), 4.1% (*Grateloupia* sp., 41/1,000), 3.8% (*Ulva* sp., 37/973) and 3.5% (*Saccharina* sp., 30/870) of all phycosphere genera (14). At the same time, these genera accounted for 76.6% (*Gelidium* sp., ± 16.5), 79.5% (*Grateloupia* sp., ± 9.2), 85.5% (*Ulva* sp., ± 5.3) and 72.8% (*Saccharina sp.*, ± 17.6) of all phycosphere bacteria (Table S1 in Additional file 3).

*Taxa with discernible host-specific and seasonal patterns*

The complete 16S rRNA amplicon dataset contained 18,434 ASVs representing genus-level host-specific phycosphere core taxa (total 51,132). Core phycosphere communities were on overall remarkably stable, whereas host-specific phycosphere taxa showed a more pronounced seasonal variability (Figs. 2C, 3, S4 in Additional file 2).

Most other phycosphere taxa appeared only in individual samples, either during single or multiple seasons. Many belonged to rarer phyla, such as *Bdellovibrionota* (*Bacteriovoracaceae* and *Bdellovibrionaceae*), which reached relative abundances of up to 1.1% (*Bdellovibrionaceae*, ± 0.9) and 2.1% (*Bacteriovoracaceae*, ± 1.3) on *Gelidium* and *Grateloupia* red macroalgal species in summer, respectively. Some clades of the *Patescibacteria,* such as *Cd.* Kaiserbacteria unc., *Saccharimonadales* unc., JGI 0000069-P22 unc. and *Absconditabacteriales* (SR1) unc. exhibited relative abundances of up to 5.0% on *Ulva* sp. green macroalgae in spring (Table S1 in Additional file 3). Further non-core families exhibited high relative abundances only in particular seasons. For example, *Shewanellaceae* were always abundant on *Ulva* sp. and *Gelidium* sp. in winter and on *Grateloupia* sp. and *Saccharina* sp. in spring. Likewise, seawater and sediment exhibited the highest relative abundances of *Shewanellaceae* in the winter and spring (Fig. S4 in Additional file 2).

*Moraxellaceae*, *Trueperaceae* and *Thiotrichaceae* belonged to the dominant phycosphere community, i.e., they were consistently present on three of the four sampled algal species. *Moraxellaceae* were consistently more abundant in spring and summer and had low relative abundances on *Saccharina* sp. brown macroalgae (Fig. S4 in Additional file 2). Conversely, *Trueperaceae* were more abundant in autumn on *Ulva* sp. green algae, and in winter to spring on *Gelidium* and *Grateloupia* red algae. Gammaproteobacterial *Thiotrichaceae* were abundant on all macroalgae except *Ulva*, where they were mostly condined to winter and spring. Also in seawater *Thiotrichaceae* abundances were highest in spring (Fig. S4 in Additional file 2).

*Diversity of cultured bacteria*

In terms of diversity, there was no significant difference between strains obtained from different macroalgal samples. Families exceeding 1% sequence abundance in at least one sample were *Flavobacteriaceae* (32.8%), *Rhodobacteraceae* (24.1%), *Sphingomonadaceae* (8.1%), *Bacillaceae* (6.8%), *Vibrionaceae* (3.1%), *Moraxellaceae* (2.0%), *Alteromonadaceae* (1.7%), *Micrococcaceae* (1.5%), *Stappiaceae* (1.5%), *Halomonadaceae* (1.2%), *Pseudoalteromonadaceae* (1.2%), *Cellvibrionaceae* (1.1%), *Staphylococcaceae* (1.1%), *Shewanellaceae* (1.1%), *Intrasporangiaceae* (1.1%) and *Colwelliaceae* (1.1%) (Table S2 in Additional file 3). More species were found on algae that did not occur in the sediment and seawater controls than vice versa (Fig. S5 in Additional file 2), including in particular members of the *Bacteroidota* (genera *Aquimarina*, *Pibocella*, *Cellulophaga*, *Dokdonia*, *Flagellimonas*, *Tenacibaculum*, *Algibacter*, *Winogradskyella*, *Maribacter*, *Croceitalea*) and *Proteobacteria* (genera *Paraglaciecola*, *Psychrobacter*, *Colwellia*, *Acinetobacter*, *Cobetia*, *Halomonas*, *Sulfitobacter*, *Sphingorhabdus*, *Labrenzia*, *Erythrobacter*, *Jannaschia*, *Tateyamaria*, *Pelagibius*, *Pseudophaeobacter*, *Altererythrobacter*, *Roseovarius*). Other algae-associated species affiliated with *Actinobacteria* (genera *Kocuria*, *Microbacterium*, *Serinicoccus*, *Ornithinimicrobium*, *Arthrobacter*, *Dietzia*) and *Firmicutes* (genera *Planococcus*, *Bacillus*, *Staphylococcus*).

Compositional differences between macroalgal samples, seawater and sediment controls were confirmed by clustering using UniFrac distances (Fig. S6a in Additional file 2) and by PCoA using Bray-Curtis distances (Fig. S6b in Additional file 2). No clear pattern was discernible with respect to algal species, as samples from different algae tended to cluster according to season rather than species. Also, the two cultivation media that were used seemed to have a negligible influence (Fig. 4). When we conducted a PCoA of 16S rRNA sequences of cultured strains together with corresponding ASVs from 16S rRNA amplicon sequencing (Fig. S2 in Additional file 2) (ANOSIM: R=0.835, p=0.001, PERMANOVA: F=9.16, p=0.001), we obtained seasonal patterns for each algae that were similar as in a PCoA of ASVs from exclusively non-core taxa (Fig. 2c) ( ANOSIM: R=0.478, p=0.001, PERMANOVA: F=3.745, p=0.001). This indicates that the majority of cultured species belonged to the more diverse non-core taxa.

*MAGs and genomes of family level core taxa*

At the family level, 645/30 (macroalgae: 589/29) draft genomes and 548/24 (macroalgae: 474/22) MAGs represented core/dominant phycosphere families (Fig. S4 in Additional file 2, Table S3 in Additional file 3). Some of the families consisted solely of genera below the 2% abundance threshold, which however collectively amounted to more than 2% of the sequences. In particular *Flavobacteriaceae*, *Sphingomonadaceae*, *Rhodobacteraceae*, and *Granulosicoccaceae* were well represented, while *Trueperaceae* and *Cd* Campbellbacteria unc. were only represented by few draft genomes and MAGs, respectively. In addition, 155 genomes representing core seawater and 72 genomes representing core sediment families were obtained.

*Clustering of draft genomes and MAGs by habitat*

In a corresponding phylogenomic tree (Fig. 5a), the majority of draft genomes and MAGs from macroalgal phycospheres were interspersed by smaller clusters of draft genomes and MAGs from seawater and, to a lesser extent, sediment samples (Fig. 5a). Based on GTDB taxonomy, the tree represented in total 42 known phyla, 78 known classes and 162 known orders (Table S3 in Additional file 3). At the genus (Fig. 3) and family (Fig. S4 in Additional file 2) levels, many taxa covered a wider range of samples and thus habitats. However, on the species level, it became apparent that most originated from a single habitat and that only a minor fraction of the species had a broad habitat range (Table S3 in Additional file 3, Fig. 5b). Clades that consisted exclusively of draft genomes tended to represent less abundant non-core taxa. Conversely, the more abundant clades tended to be represented by MAGs. For instance, 17 of the 24 most abundant phyla were exclusively represented by MAGs (Fig. 5a). These MAGs comprised 708 uncultured species (95% ANI) that were represented by a single MAGs each, 54.5% of which originated from one of the macroalgal samples (Table S3 in Additional file 3).

*Hybrid PULs*

As described in the method section futher below, we split artificially predicted long hybrid *susCD* PULs into smaller PULs (Fig. S12 in Additional file 2). 241 hybrid *susCD* PULs were initially predicted and subsequently spit into 440 single *susCD* PULs plus 103 remaining hybrid *susCD* PULs. In addition, eleven tandem repeat and hybrid *susCD* PULs were divided into eleven single *susCD* PULs, eight tandem repeat *susCD* PULs and five tandem repeat and hybrid *susCD* PULs. On overall about 22.5% of PULs were located five genes or less from a contig boundary, and 10.3% even from both boundaries (Table S4 in Additional file 3). This affected MAGs to a larger extent (56.9%) than draft genomes (13.2%). Hence, PUL predictions in genomes of cultured bacteria were more complete and reliable than those in MAGs. Consequently, the average lengths of PULs from cultured bacteria was longer than from MAGs (Figs. S15, S18 in Additional file 2).

*Incomplete susCD-like PULs*

Most predicted PULs contained the characteristic *susCD*-like gene pair along with co-occurring CAZymes. There were also PULs that lacked any obvious *susD*-like gene, such as predicted digeneaside (α-D-mannopyranosyl-(1→2)-D-glycerate) PULs [1]. We identified in total 110 PULs missing *susC*-like and 169 missing *susD*-like genes. On overall, clustering based protein sequences of *susC*-only PULs was more successful than for *susD*-only PULs, with the former clustering in a single branch of the SusC tree that featured PULs rich in CE1, CE3, or CE4 possibly targeting xylose-containing polysaccharides. Some PULs have been reported to lack *sus* genes, e.g., those targeting trehalose [1]. This may be due to corresponding *susCD* gene pairs and CAZymes genes not being encoded in one canonical locus (a limitation of the PUL prediction method) [2] [3][4].

*PUL classification based on susCD presence*

We obtained in total 718 redundant draft genomes and MAGs from *Bacteroidota* (phycosphere: 566; seawater: 98; sediment: 54, with some occurring in multiple habitats). In these genomes, 4,451 PULs were predicted (Table S6 in Additional file 3). The majority featured a single *susCD* gene pair (3,670, 82.5%), followed by hybrid *susCD* PULs (449, 10.1%), tandem *susCD* (58) and tandem/hybrid *susCD* PULs (17). Hybrid *susCD* PULs might have originated from PUL fusions, as these PULs tended to be among the longest predicted PULs (Fig. 7) harboring functionally diverse CAZyme genes. Hybrid PULs were prevalent using our prediction algorithm, especially within draft genomes from cultured strains (Fig. S18 in Additional file 2). Hybrid *susCD* PULs are relatively common among *Bacteroidota*. Even if the window width for PUL detection was adjusted down to one, still 173 hybrid *susCD* PULs were predicted (Table S4 in Additional file 3). In order to estimate the diversity of targeted glycans, we compared PUL CAZyme gene compositions harnessing substrate specificities of CAZyme families and sub-families [5]. From a CAZyme gene presence/absence matrix, we then calculated Jaccard distances [6] representing pairwise CAZymes gene and sulfatase composition dissimilarities. Based on these distances, we finally partitioned the data into 2,257 clusters (100% similarity). Out of 4,451 PULs, 68% belonged to 786 clusters of at least two PULs of identical enzyme composition. while 32% had a unique composition (Table S5 in Additional file 3).

*Candidates for GT10 family enzymes*

Functional annotations revealed that all draft genomes from sequenced strains coded for CAZymes, with on average 113 genes and an astounding maximum of 483 genes in the novel species *Fulvivirga* sp. (strains 361 and M361-1). Besides typical degradative CAZmyes (GHs: glycoside hydrolases, CEs: carbohydrate esterases, PLs: polysaccharide lyases), we also looked specifically at glycosyltransferases (GTs). GT family 10 for example includes alpha-(1,3)-fucosyltransferases (FucT) that are of particular biotechnological interest [7][8]. *Muricauda* sp. (*Flavobacteriaceae*) encoded the highest number of GT10 genes (n = 3). This and four other species with GT10 genes (n=2) were all isolated from macroalgae. All represented novel species of prevalent phycosphere bacteria. While a detailed biochemical characterization of the encoded GT10 enzymes requires additional work, the identification of such candidate genes demonstrates the functional potential preserved in our strain collection.

*PUL complexity assessment*

Prior to glycan backbone depolymerization accessory structures must be removed, which explains the complexity of some PULs. We found that the numbers and complexities of PULs derived from macroalgae and sediments were higher than those from seawater samples. In addition, the proportion of predicted secreted CAZymes, were ~4/4.6% and ~4.1/5.4% (GH/PL) higher in phycosphere and sediment bacteria than in seawater, respectively (Table S3 in Additional file 3). This suggests more synergistic degradation using shared exo-enzymes in phycosphere and sediment communities than in the water column.

The lengths and CAZyme gene richness of hybrid PULs, tandem repeat, and hybrid *susCD* PULs and tandem repeat *susCD* PULs were higher than those of single *susCD* PULs (Table S5 in Additional file 3). Complex PULs (higher CAZymes richness) mainly originated from macroalgal phycosphere and sediment bacteria. In particular, a large number of complex hybrid PULs was obtained that likely target more complex and diverse macroalgal polysaccharide substrates. It was evident that long and complex PULs are more common among tandem repeat and hybrid *susCD* PULs and tandem repeat *susCD* PULs.

*Sulfatases and acetylases in PULs*

In contrast to terrestrial plants, which contain many acetylated polysaccharides [9], glycans of marine algae are often sulfated [10] and thus require desulfating sulfatases for breakdown. However, the most frequent PULs targeting laminarin, alpha-glucans and alginate rarely contained sulfatase genes, reflecting the fact that these substrate classes are mostly unsulfated. Only 7.1% of the laminarin, 12.7% of the alpha-glucan (except for clusters 66 and 72), and 6.7% of the alginate PULs contained sulfatase genes. The abundance of laminarin and alpha-glucan PULs can be explained by their function as energy storage molecules that requires a simple, easily accessible, unsulfated structure allowing for quick mobilization of glucose monomers.

Well-known sulfated polysaccharides include galactans (agars and carrageenans) from red algae, ulvans from green algae, and fucans and fucoidans from brown algae. In particular PULs predicted to target ulvans, rhamnans, carrageenans and fucose-containing sulfated polysaccharides (FCSPs) mostly contained sulfatase genes (100%, 83.1%, 78.9%, and 78.8%, respectively). Most of these polysaccharides have persistent structural functions and thus are more complex and thioesterified [10]. As expected, the percentage of PULs with deacetylase genes (2.0%) was much lower than that with sulfatase genes (22.3%).

*Horizontal gene transfer of PULs*

We assessed the phylogeny of SusC and SusD sequences and associated PUL structures spanning at least 27 families and 70 genera of the *Bacteroidota*. PUL conservation was not always congruent with the 16S phylogeny, which may indicate frequent horizontal transfer (HGT) of PULs (Table S5). This was not only evident for laminarin PULs (Table S5 in Additional file 3, Fig. 6), but also for PULs corresponding to other polysaccharides. We also observed that CAZyme genes close to *susCD* gene pairs tended to be more conserved than those farther away. This is likely a functional effect of coupling recognition, binding and transport of oligosaccharides across the outer membrane by *susCD* with dedicated CAZymes. It might explain, why it seemed that in particular these core CAZymes are preserved in PUL LGT events. Such putative LGT was mostly predicted between members of the same class or order. We consider such events as an important driving force for microbial evolution and niche adaptation within macroalgal ephiphytic microbial communities [11].

*Phaeophyta*

*- Laminarins*

Laminarins are short chain β-1,3-linked glucans with occasional β-1,6-branches [12]. Enzymes from the GH families 5, 8, 9, 16, 17, 30, 64, 81, and 157 are involved in laminarin degradation. The backbone is usually broken down by GH16_3 endo-glucanases. PULs targeting laminarin were the most frequent of all predicted PULs (618 PULs, Table S5 in Additional file 3). This reflects the fact that laminarin is one of the most abundant macromolecules on Earth, as it acts as storage compound in brown algae and diatoms [13][14]. A large number of such PULs were also found in *Bacteroidota* genomes isolated from red and green macroalgae, which do not contain laminarin. For example, most *Aquimarina* strains had the potential to degrade laminarin regardless of origin (Table S5 in Additional file 3). Results were highly dependent on bacterial taxonomy instead of host origin.

*- Alginates*

Alginates are common in brown algae [15][16]. Alginates are linear co-polymers consisting of homopolymeric blocks of (1→4)-linked β-D-mannuronate and α-L-guluronate residues that are covalently linked in alternating sequences or blocks [1]. Alginates are anionic and bind sodium and calcium ions. Alginates were the fifth most frequently predicted PUL substrates in our dataset. They were predicted in 287 of the draft genomes, amounting to a total of 426 alginate-specific PULs. Alginate PULs encode PL6, 7, 14,15, and 17 family alginate lyases [11]. Such PULs have for example been described in numerous *Flavobacteriaceae*, such as members of the NS5 marine group, *Polaribacter*, *Aurantivirga*, and *Gramella* [1][17]. Previous studies on kelp biofilms yielded bacterial MAGs enriched in alginate degradation genes [11]. In our study, *Flagellimonas*, *Aquimarina,* and *Saprospiraceae* revealed strong degradation potentials for alginates, notably without host-specificity towards just *Saccharina* sp. brown algae.

*- Fucose-containing sulfated polysaccharides (FCSPs)*

Fucoidans occur in brown macroalgae, such as *Fucus vesiculosus*, *Laminaria* spp. (kelp) and *Macrocystis* spp. [18]. The main monomer is sulfated L-fucose, but the chemical composition of fucoidans is often complex and contains other monosaccharides (mannose, galactose, glucose, xylose, etc.), uronic acids, acetyl groups and even proteins [19]. Known fucosidases are present in the GH29, 95, 107, 141 and 151 families [19]. In a study on *Verrucomicrobia*, it was shown that FCSP degradation requires sulfatases to remove sulfate moieties before GH107 endo-fucanases can cleave the backbone into oligosaccharides. Exo-fucosidases of the GH29, 95, and 141 families subsequently hydrolyze these into fucose and other monomers [19]. It is noteworthy in this context that also MAGs obtained from kelp-colonizing bacteria were rich in enzymes for the degradation of fucoidans (CBM47, GH29, GH95) [11][15][16].

Fucoidan degradation activity has been reported in *Gammaproteobacteria* [20], *Rhodobacteraceae* [21], *Flavobacteriaceae* (*Mariniflexile fucanivorans*) [22][23], and *Verrucomicrobiota* [19][24]. We found FSCP-targeting PULs in members of the genera *Algibacter*, *Maribacter*, *Polaribacter*, *Aquimarina*, *Fulvivirga*, *Flagellimonas*, *Marinilabiliaceae*, and *Prolixibacteraceae* (Fig. 6, Table S5 in Additional file 3).

*Chlorophyta*

*- Beta-xylose-containing substrates*

Xylan is a major component of many plant cell walls. It is one of the most structurally variable polysaccharides and occurs for example as arabinoxylan (cereal grains), glucuronoxylan, acetylated and sulfated xyloglucan (marine *Chlorophyta*), or as glucuronoxylan (fruit and vegetables) [1][25][26]. Xylan-targeting PULs can contain GH8, 10, 30, 43, 51, 67, 74, or 115 families. PULs containing GH67 and 115 may target glucuronoxylan, while PULs containing GH51 may target arabinoxylan. Apart from a separate locus coding for a multi-modular GH10 xylanase, predicted xylan degradation genes in this study constituted a single cluster containing GH3, 8, 43 and 115 family genes (Table S5 in Additional file 3). In addition to GHs, there are also a large number of PULs that contain CEs. Such as, family CE7 and 17 carbohydrate esterases have been primarily characterized in terrestrial plants. They act as glucuronyl esterases and acetyl-xylan esterases that degrade lignocellulose and remove acetyl groups from hemicelluloses [27]. CE7 and 15 family enzymes from marine microbes have been characterized and their activities on xylans have been demonstrated [27][28]. In our dataset, a significant proportion of PULs encoded carbohydrate esterases of families CE1, 3, 6, 7, and 15, known to be capable of removing acetyl groups. These PULs thus may target oligosaccharides containing acetylated xylose, suggesting the presence of xylan acetylation in marine macroalgae. In addition, studies have shown that utilization of some sugars might be facilitated by acetyl xylan esterases (CE6) that are shared within macroalgal phyosphere communities [11]. These bacteria show the potential to utilize xylan. A large number of PULs targeting xylan substrates were found in bacteria in this study, as 344 isolates featured in total of 779 putative xylan-specific PULs.

*- Mannose-rich substrates*

There are two main classes of mannans, α-mannans and β-mannans with α-1,6- or β-1,4/1,3-mannan backbones, respectively. Beta-mannans have been reported in red and various green macroalga [29], where linear β-mannan seems to replace cellulose as the main cell wall glycan. Such β-mannans are for example targeted by GH26 and 130 CAZymes. Additional GH2, 3, and 88 family genes that all represent diverse functions have been reported in predicted β-mannan-targeting PULs as well [1]. In contrast, predicted α-mannan-targeting PULs have been found to feature GH38, 76, 92, 99, and 125 family CAZymes. They might be targeting α-glucomannans, such as glucuronomannan, a polysaccharide that has been reported for brown algae [15][16][30]. Most α-mannan-targeting PULs code for multiple GH92 family exo-mannosidases and at least one GH76 family endo-α-1,6-mannanase. GH92-enriched PULs are thought to potentially target α-mannose-rich N-glycosylated glycoproteins that are widespread in eukaryotes, including algae [1]. In this study, 238 genomes featured a total of 488 PULs targeting mannose-rich substrates.

*- Pectic glycans*

Pectins occur in plant cell walls and are abundant in fruits and vegetables, but have also been shown to be also a substrate of marine bacteria [31]. The two major pectins are homogalacturonan (HG) and rhamnogalacturonan-I (RGI). HG consists of α-1,4-linked D-galacturonic acid (D-GalA), while the backbone of RGI consists of repeating units of the disaccharide α-1,2-L-rhamnose (Rha)-α-1,4-D-GalA. The backbones of HG and RGI are covalently linked [32]. Members of PL1, 2, 3, 9, 10, 22, and GH28, GH105, CE8, CE12, CE13, and CE19 enzymes are involved in microbial pectin degradation [33]. In our dataset, we identified 72 PULs in 37 genomes (Table S5 in Additional file 3) featuring GH28 α-1,4-polygalacturonases, GH88 unsaturated β-glucuronyl hydrolases, family PL1, 9, 22 and 10 pectate lyases, CE8 carbohydrate esterases, and CE12 pectin methylesterases.

*- Ulvans*

Ulvans are acidic polysaccharides with a structural function in cell walls of green algae [34]. They are highly sulfated and essentially composed of rhamnose-3-sulfate, xylose, xylose-2-sulfate, glucuronic acid, and iduronic acid monomers [34]. Ulvan lyases occur in families PL24, 225, 28, 37, and 40. In our dataset 68 genomes featured in total 127 predicted ulvan PULs.

*Rhodophyta*

*- Carrageenans*

Carrageenans are sulfated galactans in marine red algae and in seagrass [35][36]. Carrageenans are mainly composed of alternating 3-linked β-D-galactopyranose (G-units) and 4-linked α-D-galactopyranose (D-units) or 4-linked 3,6-anhydro-α-D-galactopyranose (DA-units), forming a repeating disaccharide unit [37]. GH16, 82, 167, 150 and 127 family enzymes are involved in microbial carrageenan degradation [3].

Our data contained 113 PULs with characteristic carrageenase, GH110 family α-galactosidase, and sulfatase genes that likely target sulfated, galactose-rich substrates (Table S5 in Additional file 3). Some of these PULs coded for additional GH16_17, 82, 127, or 167 family CAZymes as well as predicted GH2 family β-galactosidases (Table S5 in Additional file 3). In addition we identified sulfatases specific for carrageenans in the flavobacterial genera *Zobellia*, *Aquimarina*, *Tenacibaculum*, *Flavivirga*, and *Algibacter*.

*- Agars*

Agars are jelly-like polysaccharides present in the cell walls of some red algal species [38]. Agars consists of a mixture of mainly agarose and to a lesser extent agaropectin. Agarose forms a linear polymer of repeating units of agarobiose (disaccharide of D-galactose and 3,6-anhydro-L-galactopyranose). Agaropectin consists of alternating units of D-galactose and L-galactose that are heavily modified by sulfate and pyruvate groups [38]. GH16, 50, 86, 96, 117 and 118 family enzymes are involved in microbial agar degradation. In our dataset, a total of 106 genomes featured 192 predicated agar-specific PULs.

*- Porphyrans*

Porphyrans are present in red algae. They are sulfated galactans composed of alternating 1,4-linked α-L-galactopyranose-6-sulfate (L6S) and 1,3-linked β-D-galactopyranose (G) [39]. GH16 and 86 family enzymes are involved in microbial porphyran degradation. Our dataset featured a total of 31 genomes with 31 predicted porphyran-specific PULs.

*Other polysaccharide substrates*

*- Alpha-glucans*

PULs predicted to target α-1,4-glucans, such as starch (α-1,4- and α-1,6-glucan), pullulan (α-1,4- and α-1,6-glucans), dextran (α-1,6-glucan), glycogen (α-1,4- and α-1,6-glucans) and amylose (α-1,4-glucans) were highly abundant (482 PULs, Table S5 in Additional file 3). Starch is widely found in terrestrial plants, microalgae [40], macroalgae and animals, and thus plays a central role in life as a principal store of chemical energy [41][42]. Enzymes of families GH13, 15, 31, 57, 65, 70, 71, 77, and 87 are involved in α-glucan degradation. Some PULs featured only a single GH13 and GH65 gene. These PULs likely target was structurally simple, non-branched α-1,4-glucans such as maltodextrin or amylose [1]. Our dataset featured with 596 predicted α-glucans-specific PULs.

*- Chitin*

Chitin is structurally similar to cellulose, except that the C_2_-hydroxyl (-OH) group is replaced by an acetamide group (NH-CO-CH_3_). It is thus a homopolymer of (1→4)-β-linked N-acetylglucosamine (GlcNAc) and belongs to the most abundant polysaccharides in marine habitats. Chitinases are for example present in CAZyme families GH18, 19, 20 and 23. Such chitinases frequently possess chitin-binding domains, e.g., of families CBM5, 12, and 50. Recently, chitinase-encoding genes have been identified in *Aquimarina* strains from marine sponges, corals, sediments, and seawater [43]. We found that macroalgae-associated bacteria of the genera *Aquimarina*, *Tenacibaculum*, *Carboxylicivirga* and *Formosa* contained putative chitin-PULs (Table S5 in Additional file 3), highlighting species of *Flavobacteriaceae* as source of putative novel chitinolytic enzymes. Diverse and abundant lineages within these major bacterial taxa were indeed present in our macroalgal microbiomes (Figs. 3, 6, S4 in Additional file 2).

*- Sulfated α-rhamnose-containing polysaccharides*

Sulfated rhamnans have been reported for green macroalgae [34]. Our dataset comprised 109 genomes containing a total of 219 PULs predicted to target sulfated α-rhamnose-containing polysaccharides. These PULs featured predicted GH78 family α-L-rhamnosidase genes often accompanied by GH28 family rhamnogalacturonase genes (Table S5 in Additional file 3). Some of these rhamnose-targeting PULs coded for additional predicted GH105 family rhamnogalacturonyl hydrolases, which cleave rhamnose from uronic acids. As GH78 family enzymes have also been shown to act on rhamnogalacturonans, it seems likely that these GH105-containing rhamnose-PULs target rhamnogalacturonans.

*- Peptidoglycan*

Peptidoglycan (PG) is an essential macromolecule of most eubacterial cell walls [44]. It is composed of a repeating backbone of N-acetylglucosamine (NAG) and N-acetylmuramic acid (NAM), connected by β-1→4 glycosidic bonds. Members of glycoside hydrolase families GH18, 23 (lytic transglycosylases), and 73 (β-N-acetylglucosaminidases) are involved in peptidoglycan degradation. Our dataset contained 66 genomes harboring 69 PULs predicted to target PG. These included members of *Aquimarina*, *Formosa*, *Carboxylicivirga,* and the *Saprospiraceae* that had GH23 and 73 family genes.

*- N-glycans*

Macroalgal glycans represent a significant nutrient source, and access to these host molecules appears to be important for microbial phycosphere colonization. Among these polysaccharides are eukaryotic N-glycans. The GH18 and GH63 families include endo-β-N-acetylglucosaminidases with a range of different specificities for different types of N-glycans. Some strains of *Tenacibaculum* and *Carboxylicivirga* were predicted to utilize N-glycans via a single PUL targeting high mannose N-glycans (HMNG) (Table S5 in Additional file 3). Our dataset comprised 23 genomes containing a total of 23 PULs predicted to target N-glycans.

*- Sialic acids*

GHs acting on sialic acids (GH33) and glycosaminoglycans (GH88) were enriched in macroalgae-associated genomes in this study. In total 16 genomes harboring 19 PULs could code for the capacity to tackle sialic acid oligosaccharides via PUL-associated GH33 family sialidases (Table S5 in Additional file 3).

**Supplementary Methods**

*Isolation and plate cultivation of bacteria*

Phycosphere bacteria: Complete macroalgae were cut into 9 cm^2^ pieces and rinsed thrice with sterile seawater. Afterwards 10 g of these pieces were washed with 10 ml sterile seawater (rotary shaker, 170 rpm., 30 min, 25 °C). One milliliter aliquots were diluted stepwise to 1:100,000 (sterile seawater), and 100 μl were subsequently plated and incubated (21 d, 28 °C).

Seawater samples: One milliliter seawater was diluted stepwise with 9 ml sterile seawater to 1:1,000. Aliquots of 100 μl were then plated and incubated as described above.

Sediment samples: Sediment samples of 1 g were thoroughly mixed with 9 ml sterile seawater (rotary shaker, 170 rpm., 30 min, 25 °C) and then diluted in 1:10 steps with sterile seawater to 1:10,000 [45]. Afterwards, 100 μl aliquots were plated as described [45].

Two media were used for plating, (a) modified 2216E: 18 g sea salt (Sigma-Aldrich, St. Louis, MO, USA), 1.5 g peptone, 0.3 g yeast extract, 0.3 g sodium pyruvate, 0.3 g glucose, 15 g agar, 2 g alginate, 2 g starch, 2 g carrageenan, 2 g cellulose and 0.5 mg vitamin B_12_; and (b) modified VY/2: 18 g sea salt (Sigma-Aldrich), 1 g CaCl_2_, 5 g active yeast, 0.3 g sodium pyruvate, 0.3 g glucose, 15 g agar, 2 g alginate, 2g starch, 2 g carrageenan, 2 g cellulose, and 0.5 mg vitamin B_12_ (all amounts per 0.5 L distilled water plus 0.5 L old seawater). Plating was completed within 2 h after sampling. After incubation, colony-forming units were counted with numbers ranging from 10 to 500. Colonies were selected depending on color, size, and shape. Picked colonies were purified by serial cultivation on plates with identical media. Purified strains were stored at -80 °C in sterile 1% (w/v) saline medium with 15% (v/v) glycerol.

*DNA extraction from cultured strains and environmental samples*

Cultured strains: DNA was extracted using the MiniBEST Bacteria Genomic Extraction kit v3.0 (TaKaRa Bio, Kusatsu, Shiga pref., Japan) and stored at -80 °C until use. Quality was checked by running the samples on 1% sodium boric acid agarose gels and measuring DNA concentrations using a NanoDrop 1000 spectrophotometer (Thermo Fisher Scientific, Waltham, MA, USA) based on 260/280 nm and 260/230 nm absorbance ratios. Beijing Novogene Biotechnology (Beijing, China) performed sequencing using 150 bp PE technology on an Illumina NovaSeq 6000 (Illumina, San Diego, CA, USA) with a coverage exceeding 100x. Reads were filtered as follows: (i) reads with ≥40% low-quality bases (Q ≤20) were removed, (ii) reads with ≥10% ambiguous bases were removed.

Environmental samples: Loosely attached microorganisms were removed by washing whole macroalgae thrice in flasks that were about 80% filled with sterile seawater on a shaker and collecting the washing suspensions in sterile bottles. Uncut algae fronds were then put in sterile water and the remaining bacteria were removed by ultrasonication (2x 60 s, 50-60 kHz). The bacterial suspensions were subsequently pooled with the previously obtained washing suspensions for each sample. Per sample, about 60 L of suspended bacteria were filtered through 0.2 μm pore size polycarbonate membrane filters (Millipore, Billerica, MA, USA). DNA was extracted from these filters as described elsewhere [46] with adjustments, by means of the ADX1120 Advanced Water DNA Kit (Guangdong Magigene Biotechnology Co. Ltd., Shanghai, China). For seawater, 1 L of the sample was filtered on a 0.2 μm pore size polycarbonate membrane filter (Millipore). DNA was subsequently extracted from filters using the ADX1120 Advanced Water DNA Kit (Guangdong Magigene Biotechnology), and quantified using the Invitrogen Quant-iT PicoGreen dsDNA reagent (Thermo Fisher Scientific). DNA concentrations ranged from <1 to 20 mg/mL. For sediments, DNA was extracted from 300 mg sub-samples using the Power soil DNA isolation kit (MO BIO, Carlsbad, CA, USA).

*Metagenome sequencing*

We sequenced 23 bacterial metagenomes, namely 4x *Ulva* sp., 4x *Grateloupia* sp., 4x *Gelidium* sp., 3x *Saccharina* sp., 4x surrounding seawater and 4x surrounding sediment. After DNA extraction (see previous section), metagenome libraries were prepared by sonicating the DNA to a 350 bp insert size range. DNA fragments were subsequently sequenced in paired-end mode (2 × 150 bp) on the Illumina NovaSeq 6000 platform according to the manufacturer’s instructions for metagenome analyses at Guangdong Magigene Biotechnology. A total of 1.4 Tbp (avg: 65 Gbp per metagenome) were generated (Table S1 in Additional file 3).

*Diversity analyses*

Alpha diversity metrics were calculated after rarefying the samples to 12,460 reads per sample. OTUs were clustered at ≥97% similarity. ASV richness, Simpson and Shannon's indices were calculated in R v3.5.1 ([http://www.r-project.org](http://www.r-project.org/)) using the vegan, ggplot2, ggpubr, and dplyr packages. Jaccard distances based on the presence/absence of taxa were generated using pairwise comparisons of macroalgae, seawater and sediment OTUs. Jaccard distances were obtained from principal coordinate analysis in R using the vegan, ggpubr, reshape2 and ggsci packages.

*Selection of housekeeping genes for phylogenetic analysis*

Sequences of the following ribosomal proteins were used for phylogenomic analysis of draft genomes and MAGs: S12/S23, L1, L13, L14, L16, L17, L18p, L19, L2, L20, L21p, L22, L23, L27, L27A, L28, L29, L3, L32p, L35p, L4, L5, L6, L9_C, S10, S11, S13, S15, S16, S17, S19, S2, S20p, S3_C, S6, S7, S8 and S9.

*Annotation of genes coding for sulfatases, SusC- and SusD-like proteins*

Genes coding for sulfatases, SusC- and SusD-like proteins were predicted using HMMER with the Pfam profiles PF00884 (sulfatase), PF07715, PF07980, PF12741, PF14322, and PF12771 (SusD-like), and the TIGRFAM profiles TIGR04056 TIGR01352, TIGR01778, TIGR01779, TIGR01782, TIGR01783, TIGR01785, TIGR01786, TIGR02796, TIGR02797, TIGR02803, TIGR02804, TIGR02805, TIGR04057, PF00593 (TonB-dependent receptor) with a search cutoff of E-10.

*PUL definitions used in this study*

Catabolic CAZymes often cluster in polysaccharide utilization loci (PULs). Most published and experimentally verified PULs have been found in *Bacteroidota*, where they usually contain a characteristic *susCD* gene tandem. We used a broader definition and searched loci with at least three genes coding for either CAZymes, sulfatases, SusC- or SusD-like proteins, or other TonB-dependent transporters. These loci were divided into four categories (Fig. S12a in Additional file 2): (i) PULs consisting of CAZyme genes and a *susCD* pair, (ii) CAZyme-rich gene clusters (CGC) consisting solely of CAZymes, (iii) PUL-like clusters with CAZyme genes and an encoded TonB-dependent receptor, and (iv) *susCD* loci without detectable CAZymes.In some cases, the sequence similarity of a TBDT was too low to be considered as a SusC-like or SusD-like protein. Corresponding loci were still considered as incomplete PULs [1][2].

We divided PULs into four additional categories (Fig. S12b in Additional file 2): (i) tandem repeat *susCD* PULs containing at least one tandem *susCD* pair, (ii) hybrid PULs containing multiple non-adjacent *susCD* pairs, (iii) PULs containing a single *susCD* pair, and (iv) tandem and hybrid PULs containing both, multiple non-adjacent *susCD* gene pairs with at least one tandem *susCD* gene pair.

The number of hybrid PULs increased with increasing sliding window size, whereas the number of single *susCD* gene pair PULs was only little affected. In order to minimize detection of artificial hybrid PULs, such PULs were split when five or more contiguous non-marker genes were present. The split was made amidst the non-marker genes, ensuring that each resulting part retained at least one *susCD* gene pair.

*Prediction of PUL substrates*

PULs, at least in *Bacteroidota*, are typically defined as a co-regulated operon or regulon containing a *susC*-like and *susD*-like gene pair that encode the outer-membrane glycan-import machinery, and various CAZymes that are grouped into sequence-based families in the CAZy database [47], as well as accessory proteins (e.g., sulfatases, ABC transporters, etc.). Of course, polysaccharide utilization is not always encoded in one canonical locus, but is sometimes spread out in multiple co-regulated loci [3][4]. This might be exemplified by the larger number of CAZyme-rich gene clusters (CGCs) that we found in our analyses (Fig. S13 in Additional file 2). The number of CAZymes in a PUL may correlate with the complexity of the target polysaccharide: A single CAZyme is usually responsible for the hydrolysis of a specific type of linkage. Thus, a substantial enzymatic arsenal is required to hydrolyze highly complex polysaccharides [19][48]. Moreover, the apparent redundancy in terms of CAZyme families in some PULs may relate to targeting different similar but distinct substructures of a polysaccharide. Predicting specificity based on family (sub-family) classification, however, is challenging, since glycoside hydrolase families are often polyspecific.

We used a three-step procedure to predict possible PUL substrates. First of all, we compared CAZymes gene compositions down to the sub-family level with those in the dbCAN-PUL reference database [49] and with a rough PUL classification used by Kappelmann *et al.* [1] (Table S8 in Additional file 3). When CAZyme gene compositions were identical to known PULs, the target substrate was also considered to be the same. In a second step, we analyzed endo- and exo-acting CAZymes within PULs (Table S8 in Additional file 3) and enumerated the possible substrates (Table S5 in Additional file 3), with substrates corresponding to endo-acting enzymes as possible polysaccharide backbone, and substrates corresponding to the exo-acting enzyme as possible branched chain oligosaccharides. The third step was to construct a phylogenetic tree using the SusC/D protein sequences of all *susCD* genes in a PUL, together with those published by Kappelmann [1] and Krüger [17] and those in the dbCAN-PUL database. This tree contained sequences from at least 27 bacteroidetal families from draft genomes and MAGs of this study plus their closest relatives (Table S5 in Additional file 3). We used the clustering of these sequences to infer PUL substrates. We also observed that CAZymes close to the *susCD* gene pair tended to be more conserved than those that were located farther away. Hence, we also used such conserved CAZymes in order to improve PUL clustering. Finally, we built a consensus from the three above-mentioned methods to infer the most probable substrate class.

**Code Availability**

Software used in this study:

*AntiSMASH 5.0*: <https://github.com/antismash/antismash>;

*Anvio 6.2*: https://github.com/merenlab/anvio;

*Barrnap*: https://github.com/tseemann/barrnap;

*BBDuk v35.14*: https://github.com/BioInfoTools/BBMap;

*BBTools*: <https://jgi.doe.gov/data-and-tools/bbtools>;

*BLAST*: <ftp://ftp.ncbi.nlm.nih.gov/blast/executables/blast+/LATEST>;

*CD-HIT*: <https://github.com/weizhongli/cdhit>;

*CheckM*: <https://ecogenomics.github.io/CheckM>;

*CONCOCT*: <https://github.com/BinPro/CONCOCT>;

*dbCAN2*: <http://bcb.unl.edu/dbCAN2/index.php>;

*DADA2*: <https://github.com/benjjneb/dada2>;

*dRep* : <https://drep.readthedocs.io/en/latest/>;

*DIAMOND*:<http://www.diamondsearch.org/index.php>;

*eggNOG-mapper*:<https://github.com/eggnogdb/eggnog-mapper>;

*FastANI*:<https://github.com/ParBLiSS/FastANI>;

*FastQC*:<http://www.bioinformatics.babraham.ac.uk/projects/fastqc>;

*FastTree*:<http://www.microbesonline.org/fasttree>;

*FeGenie*:<https://github.com/Arkadiy-Garber/FeGenie>;

*GTDB-Tk*: <https://github.com/Ecogenomics/GTDBTk>;

*HMMER*: <http://hmmer.org>;

*iTOL v6.5.6*: https://github.com/iBiology/iTOL;

*MAFFT*: <https://mafft.cbrc.jp/alignment/software>;

*MaxBin2*: <https://sourceforge.net/projects/maxbin2>;

*MEGAHIT*: <https://github.com/voutcn/megahit>;

*MetaBAT2*: <https://bitbucket.org/berkeleylab/metabat>;

*Prodigal*: <https://github.com/hyattpd/Prodigal>;

*Prokka*: <https://github.com/tseemann/prokka>;

*R*: <https://www.r-project.org>;

*SAMTools*: <http://www.htslib.org>;

*SPAdes*: https://github.com/ablab/spades;

*SignalP v5.0*: <https://github.com/nextgenusfs/funannotate>;

*vsearch*: https://github.com/torognes/vsearch

**References**

1. Kappelmann L, Krüger K, Harder J, Markert S, Unfried F, Becher D, et al. Polysaccharide utilization loci of North Sea *Flavobacteriia* as basis for using SusC/D-protein expression for predicting major phytoplankton glycans. ISME J. 2019;13:76–91.

2. Hemsworth GR, Déjean G, Davies GJ, Brumer H. Learning from microbial strategies for polysaccharide degradation. Biochem Soc Trans. 2016;44:94–108.

3. Ficko-Blean E, Préchoux A, Thomas F, Rochat T, Larocque R, Zhu Y, et al. Carrageenan catabolism is encoded by a complex regulon in marine heterotrophic bacteria. *Nat Commun.* 2017;8:1685.

4. Grondin JM, Tamura K, Déjean G, Abbott DW, Brumer H. Polysaccharide utilization loci: Fueling microbial communities. J Bacteriol. 2017;199:1–15.

5. Lapébie P, Lombard V, Drula E, Terrapon N. *Bacteroidetes* use thousands of enzyme combinations to break down glycans. *Nat Commun*. 2019;10: 2043.

6. Finch H. Comparison of Distance Measures in Cluster Analysis with Dichotomous Data. J Data Sci. 2021;3:85–100.

7. Petschacher B, Nidetzky B. Biotechnological production of fucosylated human milk oligosaccharides: Prokaryotic fucosyltransferases and their use in biocatalytic cascades or whole cell conversion systems. J Biotechnol. 2016;235:61–83.

8. Jost F, de Vries T, Knegtel RMA, Macher BA. Mutation of amino acids in the alpha 1,3-fucosyltransferase motif affects enzyme activity and Km for donor and acceptor substrates. Glycobiology. 2005;15:165–75.

9. Biely P. Microbial carbohydrate esterases deacetylating plant polysaccharides. Biotechnol Ad. 2012;30:1575–88.

10. Barbeyron T, Brillet-Guéguen L, Carré W, Carrière C, Caron C, Czjzek M, et al. Matching the diversity of sulfated biomolecules: Creation of a classification database for sulfatases reflecting their substrate specificity. PLoS One. 2016;11:1–33.

11. Song W, Wemheuer B, Steinberg PD, Marzinelli EM, Thomas T. Contribution of horizontal gene transfer to the functionality of microbial biofilm on a macroalgae. ISME J. 2021;15:807–17.

12. Vidal-Melgosa S, Sichert A, Francis T Ben, Bartosik D, Niggemann J, Wichels A, et al. Diatom fucan polysaccharide precipitates carbon during algal blooms. Nat Commun. 2021;12:1–13.

13. Becker S, Tebben J, Coffinet S, Wiltshire K, Iversen MH, Harder T, et al. Laminarin is a major molecule in the marine carbon cycle. Proc Natl Acad Sci. 2020;117:6599–607.

14. Alderkamp AC, Van Rijssel M, Bolhuis H. Characterization of marine bacteria and the activity of their enzyme systems involved in degradation of the algal storage glucan laminarin. FEMS Microbiol Ecol. 2007;59:108–17.

15. Deniaud-Bouët E, Hardouin K, Potin P, Kloareg B, Hervé C. A review about brown algal cell walls and fucose-containing sulfated polysaccharides: Cell wall context, biomedical properties and key research challenges. Carbohydr Polym. 2017;175:395–408.

16. Bilan MI, Grachev AA, Shashkov AS, Nifantiev NE, Usov AI. Structure of a fucoidan from the brown seaweed *Fucus serratus*. *Carbohydr Res.* 2006;341:238–45.

17. Krüger K, Chafee M, Ben Francis T, Glavina del Rio T, Becher D, Schweder T, et al. In marine *Bacteroidetes* the bulk of glycan degradation during algae blooms is mediated by few clades using a restricted set of genes. ISME J. 2019;13:2800–16.

18. Deniaud-Bouët E, Kervarec N, Michel G, Tonon T, Kloareg B, Hervé C. Chemical and enzymatic fractionation of cell walls from *Fucales*: Insights into the structure of the extracellular matrix of brown algae. Ann Bot. 2014;114:1203–16.

19. Sichert A, Corzett CH, Schechter MS, Unfried F, Markert S, Becher D, et al. *Verrucomicrobia* use hundreds of enzymes to digest the algal polysaccharide fucoidan. Nat Microbiol. 2020;5:1026–39.

20. Bakunina IY, Shevchenko LS, Nedashkovskaya OI, Shevchenko NM, Alekseeva SA, Mikhailov V V., et al. Screening of marine bacteria for fucoidanases. *Microbiology*. 2000;69:303–8.

21. Bengtsson MM, Sjøtun K, Storesund JE, Øvreas L. Utilization of kelp-derived carbon sources by kelp surface-associated bacteria. Aquat Microb Ecol. 2011;62:191–9.

22. Sakai T, Ishizuka K, Kato I. Isolation and Characterization of a Fucoidan-Degrading Marine Bacterium. Mar Biotechnol. 2003;5:409–16.

23. Barbeyron T, L’Haridon S, Michel G, Czjzek M. *Mariniflexile fucanivorans* sp. nov., a marine member of the *Flavobacteriaceae* that degrades sulphated fucans from brown algae. Int J Syst Evol Microbiol. 2008;58:2107–13.

24. Orellana LH, Francis T Ben, Ferraro M, Hehemann J-H, Fuchs BM, Amann RI. *Verrucomicrobiota* are specialist consumers of sulfated methyl pentoses during diatom blooms. ISME J. 2021;1–12.

25. Selvendran RR. Chemistry of plant cell walls and dietary fibre. Scand J Gastroenterol*.* 1987;22:33–41.

26. Bobin-Dubigeon C, Lahaye M, Guillon F, Barry JL, Gallant DJ. Factors limiting the biodegradation of *Ulva* sp cell-wall polysaccharides. J Sci Food Agric. 1997;75:341–51.

27. De Santi C, Willassen NP, Williamson A. Biochemical characterization of a family 15 carbohydrate esterase from a bacterial marine Arctic metagenome. PLoS One. 2016;11:1–22.

28. Hettiarachchi SA, Kwon YK, Lee Y, Jo E, Eom TY, Kang YH, et al. Characterization of an acetyl xylan esterase from the marine bacterium *Ochrovirga pacifica* and its synergism with xylanase on beechwood xylan. Microb Cell Fact. 2019;18:1–10.

29. Moreira LRS, Filho EXF. An overview of mannan structure and mannan-degrading enzyme systems. Appl Microbiol Biotechnol. 2008;79:165–78.

30. Wu J, Lv Y, Liu X, Zhao X, Jiao G, Tai W, et al. Structural Study of Sulfated Fuco-Oligosaccharide Branched Glucuronomannan from *Kjellmaniella crassifolia* by ESI-CID-MS/MS. J Carbohydr Chem. 2015;34:303–17.

31. Bunse C, Koch H, Breider S, Simon M, Wietz M. Sweet spheres: succession and CAZyme expression of marine bacterial communities colonizing a mix of alginate and pectin particles. Environ Microbiol. 2021;23:3130–48.

32. Coenen GJ, Bakx EJ, Verhoef RP, Schols HA, Voragen AGJ. Identification of the connecting linkage between homo- or xylogalacturonan and rhamnogalacturonan type I. Carbohydr Polym. 2007;70:224–35.

33. Luis AS, Briggs J, Zhang X, Farnell B, Ndeh D, Labourel A, et al. Dietary pectic glycans are degraded by coordinated enzyme pathways in human colonic *Bacteroides*. Nat Microbiol. 2018;3:210–9.

34. Ciancia M, Fernández PV, Leliaert F. Diversity of Sulfated Polysaccharides From Cell Walls of Coenocytic Green Algae and Their Structural Relationships in View of Green Algal Evolution*. Front Plant Sci.* 2020;11:1–15.

35. Aquino RS, Landeira-Fernandez AM, Valente AP, Andrade LR, Mourão PAS. Occurrence of sulfated galactans in marine angiosperms: Evolutionary implications. *Glycobiology.* 2005;15:11–20.

36. Michel G, Chantalat L, Fanchon E, Henrissat B, Kloareg B, Dideberg O. The ι-carrageenase of *Alteromonas* fortis: A β-helix fold-containing enzyme for the degradation of a highly polyanionic polysaccharide. *J Biol Chem.* 2001;276:40202–9.

37. Campo VL, Kawano DF, Silva DB da, Carvalho I. Carrageenans: Biological properties, chemical modifications and structural analysis-A review. Carbohydr Polym. 2009;77:167–80.

38. Zhang Y, Fu X, Duan D, Xu J, Gao X. Preparation and characterization of agar, agarose, and agaropectin from the red alga *Ahnfeltia plicata*. J Oceanol Limnol. 2019;37:815–24.

39. Qiu Y, Jiang H, Fu L, Ci F, Mao X. Porphyran and oligo-porphyran originating from red algae *Porphyra*: Preparation, biological activities, and potential applications. Food Che. 2021;349:129209.

40. Ran W, Wang H, Liu Y, Qi M, Xiang Q, Yao C, et al. Bioresource Technology Storage of starch and lipids in microalgae : Biosynthesis and manipulation by nutrients. Bioresour Technol. 2019;291:121894.

41. Øverland M, Mydland LT, Skrede A. Marine macroalgae as sources of protein and bioactive compounds in feed for monogastric animals. J Sci Food Agric. 2019;99:13–24.

42. Brányiková I, Maršálková B, Doucha J, Brányik T, Bišová K, Zachleder V, et al. Microalgae-novel highly efficient starch producers. *Biotechnol Bioeng.* 2011;108:766–76.

43. Raimundo I, Silva R, Meunier L, Valente SM, Lago-Lestón A, Keller-Costa T, et al. Functional metagenomics reveals differential chitin degradation and utilization features across free-living and host-associated marine microbiomes. Microbiome. 2021;9:1–18.

44. Sun Y, Debeljak P, Obernosterer I. Microbial iron and carbon metabolism as revealed by taxonomy-specific functional diversity in the Southern Ocean. ISME J. 2021;15:2933–46.

45. Mu DS, Liang QY, Wang XM, Lu DC, Shi MJ, Chen GJ, et al. Metatranscriptomic and comparative genomic insights into resuscitation mechanisms during enrichment culturing. Microbiome.2018;6:1-15.

46. Burke C, Kjelleberg S, Thomas T. Selective extraction of bacterial DNA from the surfaces of macroalgae. *Appl Environ Microbiol*. 2009;75:252–6.

47. Lombard V, Golaconda Ramulu H, Drula E, Coutinho PM, Henrissat B. The carbohydrate-active enzymes database (CAZy) in 2013. Nucleic Acids Res. 2014;42:490–5.

48. Reisky L, Préchoux A, Zühlke MK, Bäumgen M, Robb CS, Gerlach N, et al. A marine bacterial enzymatic cascade degrades the algal polysaccharide ulvan. Nat Chem Biol. 2019;15:803–12.

49. Ausland C, Zheng J, Yi H, Yang B, Li T, Feng X, et al. dbCAN-PUL: A database of experimentally characterized CAZyme gene clusters and their substrates. Nucleic Acids Res. 2021;49:D523–8.
