# Supplementary material for: Epiphytic common core bacteria in the microbiomes of co-located green (Ulva), brown (Saccharina) and red (Grateloupia, Gelidium) macroalgae
Source: Microbiome. 2023 Jun 1;11:126. doi: 10.1186/s40168-023-01559-1 (PMC10233909; doi:10.1186/s40168-023-01559-1)
Supplement: Supplementary file 3 — Additional file 2: Compilation of supplementary figures. Figure S1. a) Diversities of macroalgae, seawater and sediment samples as assessed by Shannon and Simpson indices as well as Good’s coverage of 16S rRNA ASVs. Statistical significance was assessed using a pairwise Wilcoxon test with Holm p-value adjustment for multiple comparisons (*, p < 0.05; **, p < 0.01; ***, p < 0.001). b) Rarefaction curves of the top 200 ASVs for all six samples and all four seasons. Figure S2. The most abundant taxa as assessed by 16S rRNA gene amplicon data. Figure S3. Phycosphere composition as assessed by 16S rRNA gene amplicon data as a function of host species and season. Figure S4. Phylogenies and abundances of the 86 most abundant families as assessed by 16S rRNA gene amplicon sequencing. Figure S5. 16S rRNA phylogenetic tree reconstruction for 202 genera that were represented by at least three cultured strains. Figure S6. Compositional differences of strains depending on sample source and season. Figure S7. Numbers of colony forming units (CFUs) per gram of sample depending on habitat and season. Figure S8. Workflow for translating GTDB taxonomic classifications to SILVA taxonomic classifications. Figure S9. Proportions of genes within 965 metagenome-assembled genomes (MAGs) and 1,618 draft genomes (DGs) with EggNOG, COG (2020), Pfam, UniProtKB, and KEGG annotations, as well as the percentage of genes lacking any functional annotation. Figure S10. CAZymes in metagenome-assembled genomes (MAGs) and draft genomes (DGs) of different phyla. Figure S11. CAZymes versus sulfatase gene frequencies in prominent phyla and families as assessed in 1,294 metagenome-assembled genomes (MAGs) and 963 draft genomes (DGs) from all six sample sources. Figure S12. Categories of loci used to find putative PULs in this study. Figure S13. Histograms of the lengths of the four loci described in Fig. S12 in metagenome-assembled genomes (MAGs) and draft genomes (DGs). Figure S14. Tree of all 159 cluster [file 40168_2023_1559_MOESM2_ESM.docx]

***Microbiome - Additional file 2***

**Epiphytic common core bacteria in the microbiomes of co-located green (*Ulva*), brown (*Saccharina*) and red (*Grateloupia*, *Gelidium*) macroalgae**

De-Chen Lu^1,2,3^, Feng-Qing Wang^2^, Rudolf I. Amann^2^, Hanno Teeling^2*^, Zong-Jun Du^1,3^*

^1^ Marine College, Shandong University, Weihai 264209, China

^2^ Max Planck Institute for Marine Microbiology, Bremen 28359, Germany

^3^ State Key Laboratory of Microbial Technology, Institute of Microbial Technology, Shandong University, Qingdao 266237, China

^*^ Corresponding authors:

Zong-Jun Du, Marine College, Shandong University, No. 180, Wenhua xilu, Weihai, Shandong Province, 264209 P. R. China, e-mail: [duzongjun@sdu.edu.cn](mailto:duzongjun@sdu.edu.cn), phone: +86 0631 5688303

Hanno Teeling, Max Planck Institute for Marine Microbiology, Celsiusstraße 1, 28359 Bremen, e-mail: [hteeling@mpi-bremen.de](mailto:hteeling@mpi-bremen.de), phone: +49 421 2028 976

**Running title:** Microbiomes of marine macroalgae

E-mail addresses and telephone numbers of all authors:

De-Chen Lu 202267000015@sdu.edu.cn +**86 0631 5688303**

Feng-Qing Wang [fwang@mpi-bremen.de](mailto:fwang@mpi-bremen.de) +49 421 2028 9390

Rudolf I. Amann [ramann@mpi-bremen.de](mailto:ramann@mpi-bremen.de) +49 421 2028 9300

Hanno Teeling [hteeling@mpi-bremen.de](mailto:hteeling@mpi-bremen.de) +49 421 2028 9760

Zong-Jun Du [duzongjun@sdu.edu.cn](mailto:%20duzongjun@sdu.edu.cn) **+86 0631 5688303**

**Conflict of interest**

The authors declare no conflict of interest.

**Supplementary Figures for this manuscript includes the following:**

Supplementary Fig. S1 to S18.

**Supplementary Figures**

**Figure S1. a)** Diversities of macroalgae, seawater and sediment samples as assessed by Shannon and Simpson indices as well as Good’s coverage of 16S rRNA ASVs. Statistical significance was assessed using a pairwise Wilcoxon test with Holm p-value adjustment for multiple comparisons (*, p < 0.05; **, p < 0.01; ***, p < 0.001). **b)** Rarefaction curves of the top 200 ASVs for all six samples and all four seasons.

​

**Figure S2. The most abundant taxa as assessed by 16S rRNA gene amplicon data.** Bars (left) represent median relative abundances of the most prominent genera. Corresponding families (right) are color-coded according to their respective phyla.

**Figure S3. Phycosphere composition as assessed by 16S rRNA gene amplicon data as a function of host species and season.** From left to right: (i) Clustering with colors representing sample sources and shapes representing seasons (UPGMA clustering of weighted UniFrac distances). Names consist of the season (1-4), macroalgal species (B=*Saccharina* sp., L=*Ulva* sp., H=*Gelidium* sp., R=*Grateloupia* sp., S=seawater, N=sediment), plus a sample replicate identifier (the numbers 1-4 represent distinct samples, and M represents the same sample that was used for metagenomics). (ii) Relative abundances of bacterial phyla. For *Proteobacteria* the most prominent proteobacterial classes *Alpha*- and *Gammaproteobacteria* are shown. (iii) Relative abundances of top-level taxa. (iv) Heatmaps of habitat-specific core taxa at the family and genus levels.

**Figure S4. Phylogenies and abundances of the 86 most abundant families as assessed by 16S rRNA gene amplicon sequencing.** Phylogeny was computed using RAxML with 1,000 bootstrap replicates. Heatmap values were obtained by converting relative abundances to Max-Min values ranging from 0-1. Samples are denoted by an initial capital letter specifying the sample (H=*Grateloupia* sp., R=*Gelidium* sp., L=*Ulva* sp., B=*Saccharina* sp., S=seawater, N=sediment), followed by a number representing season (1=autumn, 2=winter, 3=spring, 4=summer). Solid squares to the right of the heatmaps represent core families of corresponding samples. Circles and triangles represent overall core and dominant phycosphere families. The following six numerical columns represent the corresponding numbers of genomes and MAGs that were obtained.

**Figure S5. 16S rRNA phylogenetic tree reconstruction for 202 genera that were represented by at least three cultured strains.** The tree was calculated using RAxML with 1,000 bootstrap replicates. Representative full lengths 16S rRNA gene sequences for each genus were selected randomly from corresponding cultured strains. From inside to outside: (i) heatmap representing relative abundances of cultured genera in all samples (H=*Gelidium* sp., R=*Grateloupia* sp., L=*Ulva* sp., B=*Saccharina* sp., S=seawater, N=sediment); (ii) heatmap representing abundances derived from 16S rRNA amplicon sequencing; (iii) indication of core community members by solid squares for the four algae (red, green, brown), seawater (blue) and sediments (black); (iv) numbers of obtained cultured strains; (v) bars representing the percentage of new species.

**Figure S6. Compositional differences of strains depending on sample source and season.** **a)** (left) samples were grouped by weighted UniFrac distances using Ward linkage (dendrogram) between sample sources, (right) corresponding richness (showing median and inter-quartile ranges, OTU level) for each group. **b)** Principal coordinate analysis plots of Bray-Curtis similarities of samples and seasons calculated from unweighted UniFrac distances for macroalgal samples (n=52), surrounding seawater (n = 7), and surrounding sediment (n = 8). Colors correspond to sample sources and shapes to seasons. Sizes represent the two media that were used. Results of PERMANOVA and ANOSIM tests for significance between groups are shown for each plot.

**Figure S7. Numbers of colony forming units (CFUs) per gram of sample depending on habitat and season.** Depicted are numbers obtained with both media, modified marine modified VY/2 (salmon) and 2216E (turquoise) medium for samples of *Gelidium* sp. (red algae), *Grateloupia* sp. (red algae), *Ulva* sp. (green algae), *Saccharina* sp. (brown algae), sediment (5 meters underwater), and seawater (-0.1 to -0.5 m water depth). The average CFU numbers obtained from macroalgae ranged from 5.5 × 10^5^ CFU g^-1^ (modified VY/2 medium) to 5.8× 10^5^ CFU g^-1^ (modified 2216E medium). The average CFU numbers obtained from seawater and sediment samples were lower by about three and two orders of magnitude, respectively.

**Figure S8. Workflow for translating GTDB taxonomic classifications to SILVA taxonomic classifications.**

**Figure S9. Proportions of genes within 965 MAGs and 1,618 draft genomes (DGs) with EggNOG, COG (2020), Pfam, UniProtKB, and KEGG annotations, as well as the percentage of genes lacking any functional annotation.**

**Figure S10. CAZymes in MAGs and draft genomes (DGs) of different phyla.** Pie charts represent relative proportions of CAZyme classes except for GTs (glycosyltransferases) - GHs (glycoside hydrolases), PLs (polysaccharide lyases), CEs (carbohydrate esterases), AAs (auxiliary activities), and CBMs (carbohydrate-binding modules). The numbers of MAGs and DGs are indicated in parentheses. Box plots represent distributions of absolute (left) and relative (right) CAZyme numbers. Averages are represented as blue dots.

**Figure S11**. **CAZymes versus sulfatase gene frequencies in prominent phyla and families as assessed in 1,294 MAGs and 963 draft genomes (DGs) from all six sample sources.** MAGs are represented by squares and DGs by circles, with border colors representing families and fill colors representing sample types. Circle sizes correspond to genome sizes. Detailed information is provided in Table S3 in Additional file 3.

**Figure S12. Categories of** **loci used to find putative PULs in this study.** **a)** locus with only a *susCD* gene pair, CAZyme-rich gene cluster, PUL-like locus, conventional PUL. **b)** Sub-classification of PULs according to *susCD* gene arrangements.


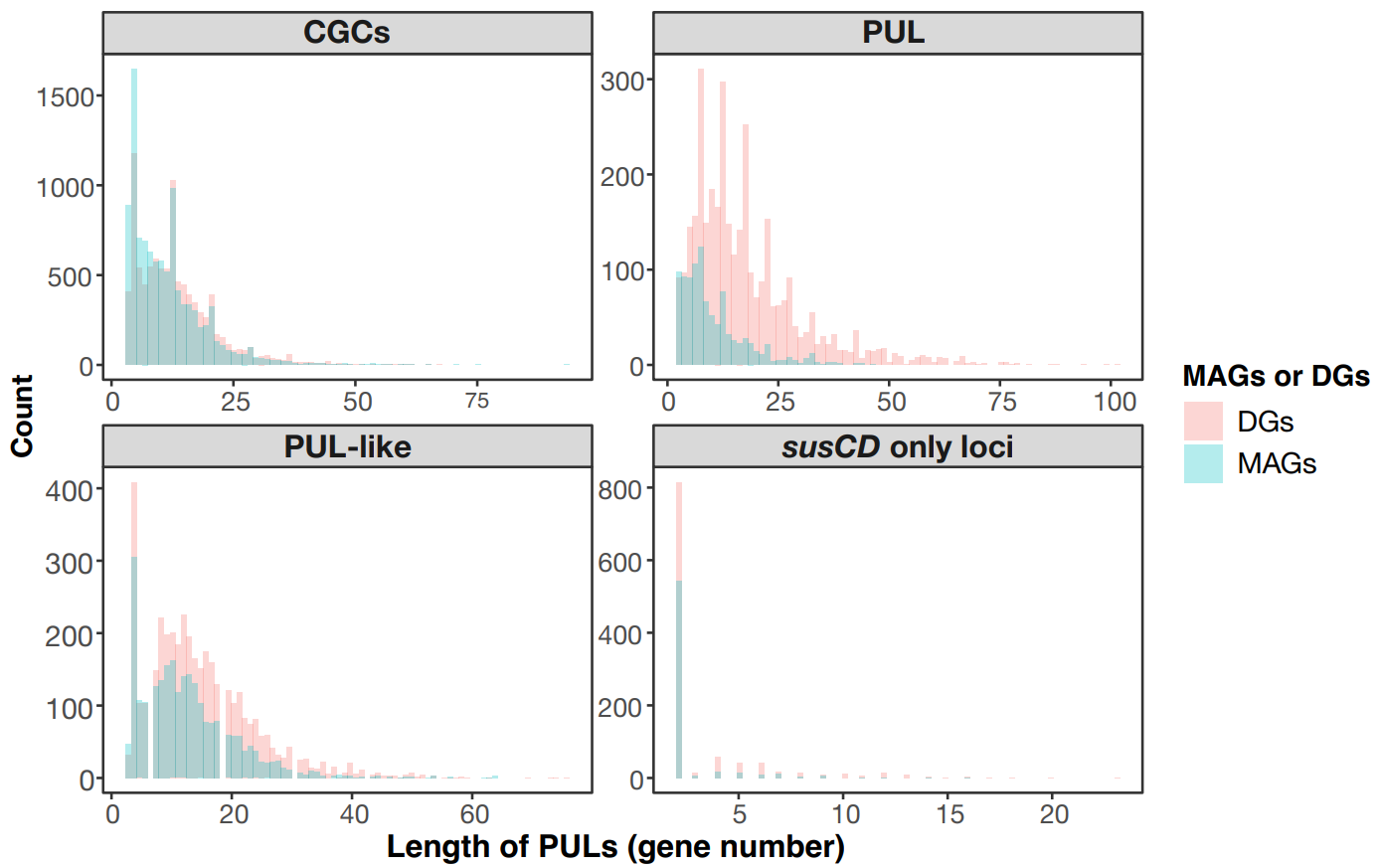


**Figure S13. Histograms of the lengths of the four loci described in Fig. S12 in MAGs and draft genomes (DGs).**

**Figure S14.** **Tree of all 159 clusters derived from 3,769 PUL-associated SusC-like protein sequences from *Bacteroidota* MAGs and draft genomes (inside to outside):** (i) cluster identifier, (ii) the number of SusC-like proteins, (iii) pie charts of corresponding samples sources, (iv) substrate predictions (short form).

**Figure S15. Basic quality metrics of the 1,619 MAGs and 965 draft genomes (DGs). Box-plots (A-E) show the minimum value, first quartile, median, third quartile and maximum value.** Of the in total 2,584 genomes, 1,506 had >90% completeness and <5% contamination, 1,023 of which contained complete rRNA operons plus at least 18 of the standard tRNAs. Details are provided in Table S3 in Additional file 3.

**Figure S16. Biosynthetic gene cluster (BGC) sizes in genomes from distinct phyla.** Four BGC categories were defined based on the distance between BGCs (see below). Each point in the box-plots represents one BGC. Red dots represent BGCs at contig edges, while blue dots represent BGCs within contigs. Numbers in parentheses refer to the number of genomes, and the corresponding total number of BGCs. Single: candidate cluster with only one BGC; chemical hybrid: candidate cluster containing BGCs sharing cluster-defining CDS/genes/gene products; interleaved: candidate clusters containing BGCs which do not share cluster-defining CDS features, but whose core locations overlap; neighboring: candidate clusters containing BGCs that do not match either chemical or interleaved variants, but transitively overlap in their neighborhoods.

**Figure S17. Clustering of biosynthetic gene clusters (BGCs) according to sample type and phylogeny.** **a)** BGC types across habitats for MAGs and draft genomes (DGs). Numbers in the parentheses represents the numbers of MAGs or DGs and the corresponding sum of BGCs. **b)** Proportions of BGCs in MAGs and DGs obtained from all six sample sources. Numbers in parentheses correspond to the total numbers of BGCs.

**Figure S18. Sizes of PULs and PUL-like loci in genomes from distinct *Bacteroidota* families (categories: hybrid *susCD*, single *susCD*, tandem repeat *susCD*, and tandem repeat plus hybrid *susCD* PULs).** Each point in the box-plots represents one PUL. Red dots represent loci at contig edges, whereas blue dots represent loci within contigs. Numbers in parentheses correspond to the number of genomes and the total number of corresponding PULs. DGs: draft genomes.
